# Role of PI3K/AKT/MAOA in glucocorticoid‐induced oxidative stress and associated premature senescence of the trabecular meshwork

**DOI:** 10.1111/acel.14452

**Published:** 2024-12-17

**Authors:** Pengyu Zhang, Nan Zhang, Yixin Hu, Xizhi Deng, Min Zhu, Cheng Lai, Wen Zeng, Min Ke

**Affiliations:** ^1^ Department of Ophthalmology Zhongnan Hospital of Wuhan University Wuhan Hubei China

**Keywords:** cell aging, glaucoma, glucocorticoid, mitochondria, oxidative stress, PI3K/AKT/MAOA, trabecular meshwork

## Abstract

The oxidative stress‐induced premature senescence of trabecular meshwork (TM) represents a pivotal risk factor for the development of glucocorticoid‐induced glaucoma (GIG). This study aimed to elucidate the pathogenesis of TM senescence in GIG. MethodsIntraocular pressure (IOP), transmission electron microscopy and senescence‐associated protein expression in TM were evaluated in GIG mice. Protein expression of phosphoinositide‐3‐kinase regulatory subunit 1 (PIK3R1) and monoamine oxidase A (MAOA), phosphorylation of AKT were quantified. ROS and mitochondrial superoxide levels were measured to evaluate cellular oxidative stress. Cell cycle analysis, β‐galactosidase staining, senescence‐associated protein expression were employed to assess the aging status of primary human trabecular meshwork cells (pHTMs). ResultsmRNA‐seq and KEGG analysis indicating PI3K/AKT pathway as a key regulator in TM of GIG. PI3K inhibitor significantly prevented IOP elevation and abnormal mitochondrial morphology of TM in the GIG mouse model. PI3K inhibitor or selective silencing of *PIK3R1* alleviated dexamethasone (DEX)‐induced oxidative stress, also mitochondrial dysfunction, inhibiting MAOA expression in pHTMs. The same phenomenon was observed in the GIG models with inhibition of MAOA. Further KEGG analysis indicates that cellular senescence is the key factor in the pathogenesis of GIG. TM senescence was observed in both GIG mouse and cell models. Inhibition of the PI3K/AKT/MAOA pathway significantly alleviated DEX‐induced premature cellular senescence of TM in GIG models. Glucocorticoids activated the PI3K/AKT/MAOA pathway, leading to mitochondrial dysfunction, oxidative stress, and premature aging in TM, elevating IOP. This mechanism could be associated with the onset and progression of GIG, providing a potential approach for its treatment.

## Graphical abstract

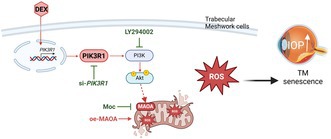

**Abbreviations**
ANOVA: analysis of variance
DEX: dexamethasone
GCs: glucocorticoids
GIG: glucocorticoid‐induced glaucoma
IOP: intraocular pressure
MAOA: monoamine oxidase A
Moc: moclobemide
mtROS: mitochondrial ROS
pHTMs: primary human trabecular meshwork cells
PIK3R1: phosphoinositide‐3‐kinase regulatory subunit 1
qPCR: quantitative polymerase chain reaction
ROS: reactive oxygen species
Sal‐β‐gal: senescence‐associated β‐galactosidase
SD: standard deviation
TEM: transmission electron microscopy
TM: trabecular meshwork
TMRE: tetramethylrhodamine ethyl ester perchlorate
WB: western blot
Ψm: mitochondrial membrane potential

## 1 INTRODUCTION

Glucocorticoids (GCs) are widely used for their anti‐inflammatory and immunosuppressive properties, yet prolonged use can lead to various side effects (Vandewalle et al., 2018), including glucocorticoid‐induced glaucoma (GIG) (Caplan et al., 2017; Patel et al., 2018). GIG manifests as a secondary form of glaucoma (Patel et al., 2023), characterized by elevated intraocular pressure (IOP), irreversible optic nerve damage, and vision loss, impeding the widespread and safe application of GCs in clinical practice (Patel et al., 2019). While the molecular mechanisms underlying the pathogenesis of GIG are not fully understood, recent research on GIG indicates that GCs trigger senescence‐related changes in transformed human trabecular meshwork (TM) cells by inducing oxidative stress (Li et al., 2023). Aging is one of the primary risk factors for glaucoma. Age‐related changes in conventional outflow tissues, such as increased cellular senescence and stiffness of TM (Li et al., 2024), leading to elevated IOP (Fini et al., 2017). Oxidative stress represents a potential mechanism that underlies TM cellular senescence in glaucoma (Yu et al., 2010). Nevertheless, the exact mechanism through which GCs induce oxidative stress and cellular senescence in TM and IOP elevation remains ambiguous.

The TM, located in the anterior chamber angle of the eye, is a pressure‐sensitive tissue responsible for approximately 80% of aqueous humor outflow (Mzyk et al., 2022; Yarishkin et al., 2021). It regulates the outflow channel resistance by remodeling the actin cytoskeleton or modifying the extracellular matrix through matrix metalloproteinase activity (Keller & Peters, 2022; Yang et al., 2016). Various factors, such as oxidative stress, mitochondrial dysfunction, DNA damage, telomere dysfunction, oncogene activation, and organelle stress, can induce premature cellular senescence, significantly affecting TM function and morphology and increasing susceptibility to glaucoma (Di Micco et al., 2021; Keller & Peters, 2022; Wang et al., 2018).

The oxidative stress theory posits that cumulative damage induced by reactive oxygen species (ROS) may lead to cellular senescence and functional decline (Forman & Zhang, 2021; Luo et al., 2020). Mitochondrial electron transport chain is the main source of ROS generation in cells, and the efficiency of ROS generation is related to the activity of monoamine oxidase A (MAOA) (Antonucci et al., 2021; Di Sante et al., 2023). Besides ROS, mitochondrial superoxide serves as an indicator of cellular oxidative stress levels (Kuznetsov et al., 2022). Oxidative stress triggers premature cellular aging, which in turn affects cellular oxidative stress states (Faraonio, 2022). Research indicates that the buildup of p16 and p21 in the TM of glaucomatous eyes is linked to the accumulation of ROS during aging, further confirming the strong association between oxidative stress and cellular senescence (Chhunchha et al., 2017; Pulliero et al., 2014).

In this study, the dexamethasone (DEX)‐induced GIG model was used to investigate the effect of GC on TM aging, revealing the important role of PI3K/AKT/MAOA pathway. High‐throughput mRNA sequencing revealed PI3K/AKT's pivotal role. Inhibiting PI3K/AKT prevented DEX‐induced oxidative stress and senescence both in vitro and in vivo. MAOA contributes to DEX‐induced mitochondrial dysfunction and aging. In summary, DEX activates PI3K/AKT, which upregulates MAOA, thereby accelerating TM aging and IOP elevation. The findings offer insights into glaucoma prevention and treatment strategies.

## 2 MATERIALS AND METHODS

### 2.1 Animals

Adult C57BL/6J mice (both male and female, 6–8 weeks old) were purchased from the Animal Center of Wuhan University. Animal procedures adhered to ARVO Statement guidelines for Ophthalmic and Vision Research and were approved by the Institutional Animal Care Committee of Wuhan University (WP20230097). Mice were housed in a controlled environment at 20°C–25°C with a 12‐h light/12‐h dark cycle and free access to food and water. Mice were randomly assigned to different treatment groups. The GIG mouse model was established as previously described (Patel et al., 2017). Briefly, DEX‐acetate (DEX‐Ace, A45645, OKA) was injected into the right eye of each mouse once a week (10 mg/mL, 10 μL/eye). Only mice with an IOP increase of ≥4 mmHg above baseline were included in this study.

### 2.2 Cells

Primary human trabecular meshwork cells (pHTMs) were isolated from postmortem donor eyes following the “Declaration of Helsinki” guidelines and the Guidelines of the Medical Ethics Committee of Zhongnan Hospital of Wuhan University. Cell isolation and identification procedures were based on previously established protocols (Artzatbanov et al., 1987). pHTMs from five donors aged 18, 31, 42, 59, and 69 years without ocular disease history were utilized. Myocilin and Matrix Gla Protein (MGP) were used for cell identification (Figure S1a) as previously described (Gould et al., 2004; Keller et al., 2018). After treating pHTMs with 100 μM DEX for 72 h, the expression of myocilin was detected through quantitative polymerase chain reaction (qPCR) and Western blot (WB) to confirm glucocorticoid responsiveness (Figure S1b,c). pHTMs were cultured in DF12 medium (Hyclone, USA) containing 10% fetal bovine serum (Gibco, USA) and 1% penicillin/streptomycin (Beyotime Biotechnology, China) (Keller et al., 2018), at 37°C with 5% CO_2_, with medium change every 24 h. Passages 4–6 were used for further experiments.

### 2.3 Drugs and cellular treatment

LY294002‐treated group mice received intraperitoneal injections of LY294002 (50 mg/kg; MedChenExpress, New Jersey, USA) once weekly for 8 weeks. Moclobemide‐treated group mice received intraperitoneal injections of moclobemide (40 mg/kg; MedChenExpress, New Jersey, USA) once every 2 days for 8 weeks. Control mice were injected with sterilized phosphate‐buffered saline (PBS), similar to the LY294002‐treated group. For ex vivo experiments, a GIG cell model was established using DEX (D4902, Sigma‐Aldrich, Darmstadt, Germany, 100 nM) for 72 h. LY294002 (50 μM; MedChenExpress, New Jersey, USA) or Moclobemide (50 μM; MedChenExpress, New Jersey, USA) treated the cells for 72 h. LY294002/Moclobemide and DEX were co‐added in the medium of pHTMs.

### 2.4 IOP

Following the induction of anesthesia using 5% isoflurane (NDC 66794–017‐25; Bethlehem, PA, USA), the intraocular pressure (IOP) of the mice was determined by utilizing a rebound tonometer (Tonolab Colonial Medical Supply in Londonderry, NH) within 2–3 min of anesthetic exposure (Wang et al., 2005). Each week, the IOP measurements were consistently taken by the same researcher, specifically between the hours of 8 a.m. and 10 a.m. The recorded values were derived from an average of three repeated measurements per animal at each time point.

### 2.5 Western blot analysis

To assess protein changes in TM tissue, we collected scleral rings (mainly trabecular meshwork with less sclera and cornea) from dissected mouse eyeballs for WB, using the same protein extraction method as before and removing as much of the sclera and cornea as possible at dissection (Fan et al., 2020). Cell samples were lysed with RIPA lysis buffer containing protease (1:100) and phosphatase inhibitor (1:100) cocktail (Santa Cruz, TX, USA) for 40 min on ice. Protein concentration was determined using a BCA Protein Assay Kit (Beyotime, Shanghai, China). Fifty micrograms of protein from each sample were separated on 10%–15% sodium dodecyl‐sulfate polyacrylamide gel electrophoresis gels and transferred to a 0.45 μm pore size polyvinylidene fluoride membrane (GE Healthcare Life Sciences, Massachusetts, USA). The membrane was blocked with 5% bovine serum albumin at 25°C for 2 h and incubated with primary antibodies (Data S1) at 4°C overnight. Thereafter, the membranes were cultivated with goat anti‐rabbit IgG secondary antibody for another 2 h at 25°C. Membranes were developed with enhanced chemiluminescence reagent using Tanon 5200 ECL imaging system (Tanon, China). Protein expression levels were semi‐quantified using ImageJ software (version 1.52). GAPDH was used as the loading control.

### 2.6 Transmission electron microscope (TEM)

For TEM testing, TM tissue from mice was fixed with Ito fixative, postfixed in osmium tetroxide, and dehydrated as previously described (He et al., 2019). After soaking the specimen in a mixture of epoxy propane and resin for 1 h, it was embedded in fresh resin and polymerized at 60°C for 24 h. Ultrathin sections (50–70 nm) were cut using an ultramicrotome (Leica UC7, Leica, Germany) and placed on copper grids. These sections were subsequently incubated with saturated uranyl acetate for 15–30 min to enhance contrast. Finally, the slices were examined using a Hitachi HT7700 120 kv transmission electron microscope (Hitachi High‐Technologies Corporation, Japan).

### 2.7 siRNA transfection

Transfection of pHTMs with siRNA followed the manufacturer's protocol using RFectPM siRNA/miRNA Transfection Reagent (Changzhou Bio‐generating Biotechnology Corp, Jiangsu, China). Cells at 30%–40% confluence were transfected with a mixture of RFect and siRNA against *PIK3R1* (20 nM; si‐*PIK3R1*: 5′‐GUACGAGAUGCGUCUACUATT‐3′, 5′‐UAGUAGACGCAUCUCGUACTT‐3′) or scrambled small‐interfering RNA as a negative control (Tsingke Biotechnology, Shanghai, China) diluted in Opti‐MEM (Gibco, Thermo Fisher Scientific, MA, USA) for 8 h. The medium was then changed, and cells were further incubated for 48 h. The success of gene knockout was confirmed by qPCR and Western blot analysis, the knockdown efficiency of si‐PIK3R1 was about 50% at both the RNA and protein levels in pHTMs (Data S1).

### 2.8 MAOA overexpression plasmid transfection

Transfection of pHTMs was transfected using Lipofect5000 plasmid/DNA transfection reagent (Changzhou Bio‐generating Biotechnology Corp, Jiangsu, China) according to the manufacturer's protocol. Under the confluence condition of 60%–80%, the overexpression group was transfected with a mixture of MAOA overexpression plasmid (Tsingke Biotechnology, Shanghai, China) and Lipofect5000 with Opti‐MEM (Gibco, Thermo Fisher Scientific, MA, USA). The control plasmid (Tsingke Biotechnology, Shanghai, China), and Lipofect5000 diluted with Opti‐MEM was used as a negative control and transfection lasted for 8 h. Then, the culture medium was changed, and the cells were further incubated for 48 h. Successful transfection was confirmed by Western blot analysis (Figure 4c).

### 2.9 Intracellular ROS test

In each pHTMs group, 0.5 mL of a diluted dichloro‐dihydro‐fluorescein diacetate probe (10 μM, S0033S, Beyotime, China) was added and incubated at 37°C for 20 min. The cells were washed three times with a 37°C culture medium. Fluorescence measurement was conducted using a fluorescence microscope (Olympus, Japan). The results were analyzed using ImageJ (version 1.52).

### 2.10 Mitochondrial ROS measurement

The levels of mitochondrial ROS (mtROS) were detected using MitoSOX™Red (M36008; USA). pHTMs were washed three times with PBS, incubated with 5 μM MitoSOX™Red at 37°C in the dark for 15 min, and washed again three times with PBS. MtROS were visualized under a microscope at 20‐fold magnification (Olympus, Japan) and analyzed using ImageJ (version 1.52).

### 2.11 Senescence‐associated β‐galactosidase test

The cells were inoculated onto a six‐well plate and treated for 72 h. After fixation at room temperature for 15 min, they were stained using a senescence‐associated β‐galactosidase (Sal‐β‐gal) staining kit (#9860, Cell Signaling Technology, Boston, United States), according to the manufacturer's instructions. One milliliter of β‐galactosidase dye solution was added to each hole and was incubated at 37°C for 16 h with proper sealing to prevent liquid evaporation. Subsequently, the cells were observed at 20‐fold magnification under a microscope (Olympus, Japan).

### 2.12 Cell cycle flow cytometry

The cells were seeded onto a six‐well plate and treated for 72 h. After treatment, pHTMs in each group were trypsinized, centrifuged at 1000*g* for 3–5 min, and precipitated as per the instructions of the manufacturer. The cells were resuspended with 1 mL of ice‐cold PBS and centrifuged, and the supernatant was carefully aspirated. The cells were then gently tapped off, fixed with ice‐cold 70% ethanol, and stored at 4°C for 12 h. After centrifugation at 1000*g* for 5 min, washing with ice‐cold PBS, and another centrifugation, the cells were stained with propidium iodide (PI) staining solution (0.5 mL/well; Cell Cycle and Apoptosis Detection Kit, C1052, Beyotime, Shanghai, China) for 30 min at 37°C in the dark. Stained cells were analyzed using a flow cytometer (CytoFlexS, Beckman, United States). The results were analyzed using FlowJo (version 10.8.1).

### 2.13 Statistics

Data analysis was performed using GraphPad Prism 9.0. Results are presented as mean ± standard deviation (SD) from multiple independent experiments. Unpaired Student's t‐test was used for comparison between two groups, while one‐way analysis of variance (ANOVA) with Tukey's correction assessed differences among multiple groups. Statistical significance was set at *p* values <0.05 (*p < 0.05, **p < 0.01, ***p < 0.001, ****p < 0.0001).

## 3 RESULTS

### 3.1 GCs activated PI3K/AKT pathway in TM

To delve into the underlying mechanisms of the pathogenesis of GIG, RNA sequencing was conducted on DEX‐treated and control pHTMs. Significant differences in PI3K/AKT, cell cycle and p53 signaling pathways between DEX‐treated and control pHTMs were revealed by KEGG enrichment analysis (Figure S3a). Further WB experiments confirmed an upregulation in the phosphorylation levels of AKT (p‐AKT‐S473, p‐AKT‐T308) in DEX‐treated pHTMs compared to control cells (Figure S3b). These results indicate that DEX induces the activation of the PI3K/AKT pathway, validating the findings of previously reported studies (Shan et al., 2017).

To identify the key genes involved in the pathogenesis of GIG within the PI3K/AKT pathway, we compared our data with the GSE16643 (Nehme et al., 2009), the GSE37474, and the GSE124114 data sets (Faralli et al., 2019) from the GEO database. We screened for DEGs using a *p*‐value threshold of <0.05 and a fold change criterion of >0.8 or <−0.8. A gene associated with the PI3K/AKT pathway with the same change trend (*PIK3R1*) was screened (Figure S3c). *PIK3R1* encodes the major regulatory subunit of class I PI3K, p85α, which regulates the catalytic activity of P110α kinase, impacting the PI3K/AKT pathway (Tsay & Wang, 2023). The subsequent qRT‐PCR and WB results confirmed that both mRNA and protein expression of PIK3R1 elevated after DEX treatment in pHTMs (Figure S3d,e), indicating that DEX induces overexpression of PIK3R1, thus activating the PI3K/AKT pathway in pHTMs.

### 3.2 The inhibition of PI3K/AKT pathway alleviated GIG progression and mitochondrial damage in TM

**FIGURE 1:**
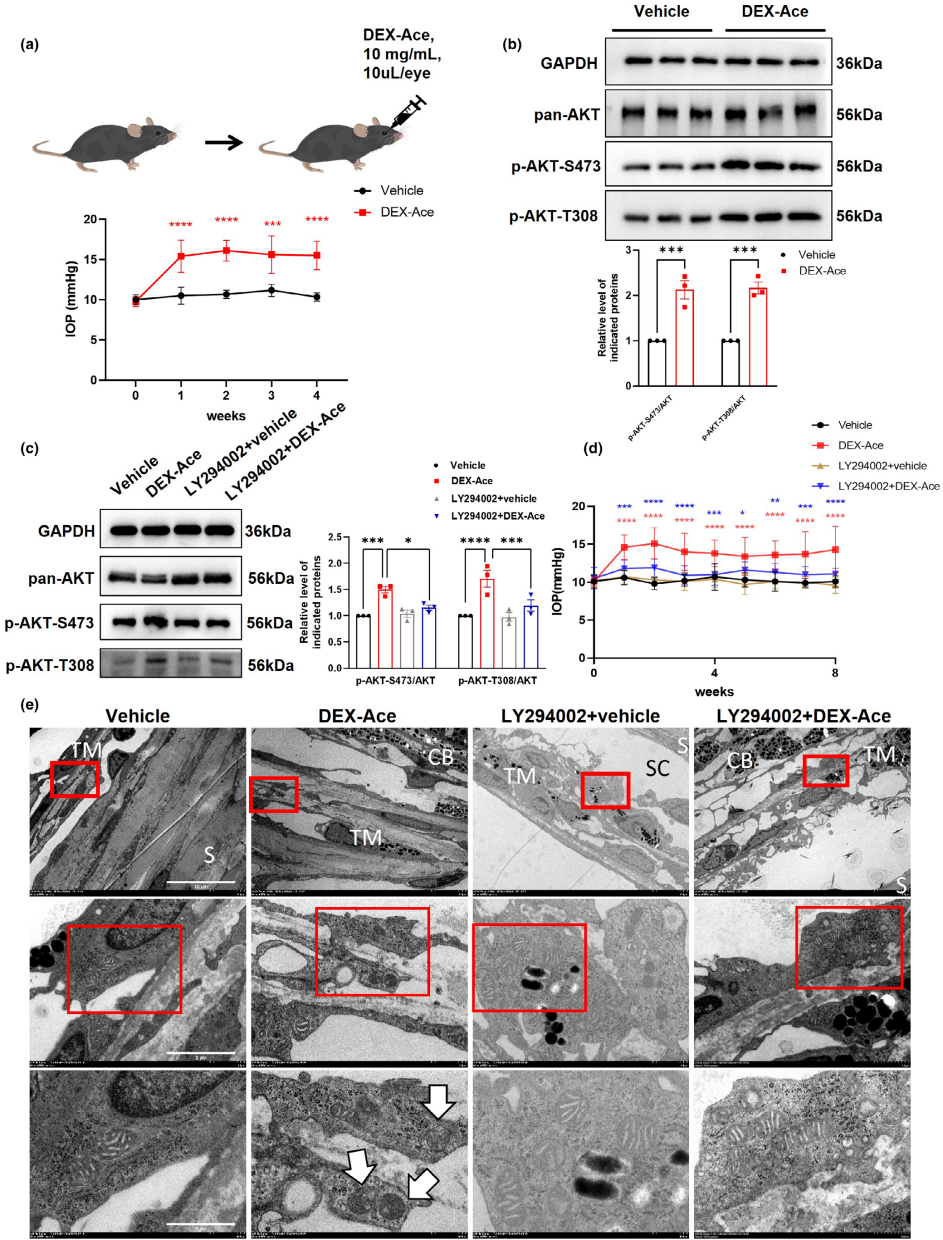
Effect of inhibition of the PI3K‐AKT pathway on GIG progression in the GIG mouse model. (a) Schematic diagram depicting the construction of the GIG mouse model and the IOP elevation in GIG mice (red asterisk: Vehicle vs. DEX‐Ace; *n* = 10 mouse eyes). (b) Protein expression levels of pan‐AKT, p‐AKT‐S473, and p‐AKT‐T308 in TM of vehicle and DEX‐Ace groups (*n* = 3 mouse eyes). (c) Protein expression levels of pan‐AKT, p‐AKT‐S473, and p‐AKT‐T308 in TM of vehicle, DEX‐Ace, LY294002 + vehicle, and LY294002 + DEX‐Ace groups (*n* = 3 mouse eyes). (d) LY294002 (PI3K inhibitor; 50 mg/kg, once weekly for 8 weeks) reduced IOP in the GIG mouse model (red asterisk: Vehicle vs. DEX‐Ace; blue asterisk: DEX‐Ace vs. LY294002 + DEX‐Ace; *n* = 10 mouse eyes). (e) Representative images of mitochondrial morphology in the TM region from each group captured by electron microscopy. White arrows: Damaged mitochondria in the DEX‐Ace‐treated group (*n* = 3 mouse eyes). Scale bar = 10 μm (upper images)/2 μm (middle images)/1 μm (lower images). CB, ciliary body; S, Sclera; SC, Schlemm's canal; TM, Trabecular meshwork. Data are presented as mean ± SD. Unpaired *t*‐test (a, b). One‐way ANOVA followed by Tukey's test (c, d). *p < 0.5, **p < 0.01, ***p < 0.001, ****p < 0.0001. All experiments were biologically replicated at least three times.

We developed a GIG mouse model by administering subconjunctival injections of DEX‐Ace (10 mg/mL, 10 μL/eye, once a week) for 8 weeks as previous report, mimicking the pathology of GIG (Deng et al., 2024; Zeng et al., 2020). As shown in Figure 1a, IOP increased after the first DEX‐Ace injection compared to baseline and remained elevated throughout the treatment period. Consistent with the sequencing results, higher levels of AKT phosphorylation were detected in the TM of GIG mice, suggesting activation of the PI3K/AKT pathway in the TM of GIG mice (Figure 1b).

To further explore the role of PI3K/AKT pathway in GIG, mice were intraperitoneally injected with LY294002 (a PI3K inhibitor; 50 mg/kg, once weekly for 8 weeks) in the GIG model. Treatment with LY294002 significantly inhibited AKT phosphorylation in TM (Figure 1c). Additionally, the PI3K inhibitor prevented the IOP elevation in GIG (Figure 1d), suggesting that inhibition of PI3K/AKT pathway partially delayed the progression of GIG.

Further observation of TM using TEM revealed alterations in mitochondrial morphology in GIG. Compared to the control group, mitochondria in the TM of DEX‐Ace‐treated mice exhibited increased ultrastructural changes, such as increased mitochondrial fragmentation and damage, suggesting that mitochondria are crucial to GIG pathology (Figure 1e). Conversely, the administration of LY294002 partially mitigated mitochondrial ultrastructural damage (Figure 1e).

### 3.3 Inhibition of the PI3K/AKT pathway attenuates DEX‐induced oxidative stress in pHTMs

**FIGURE 2:**
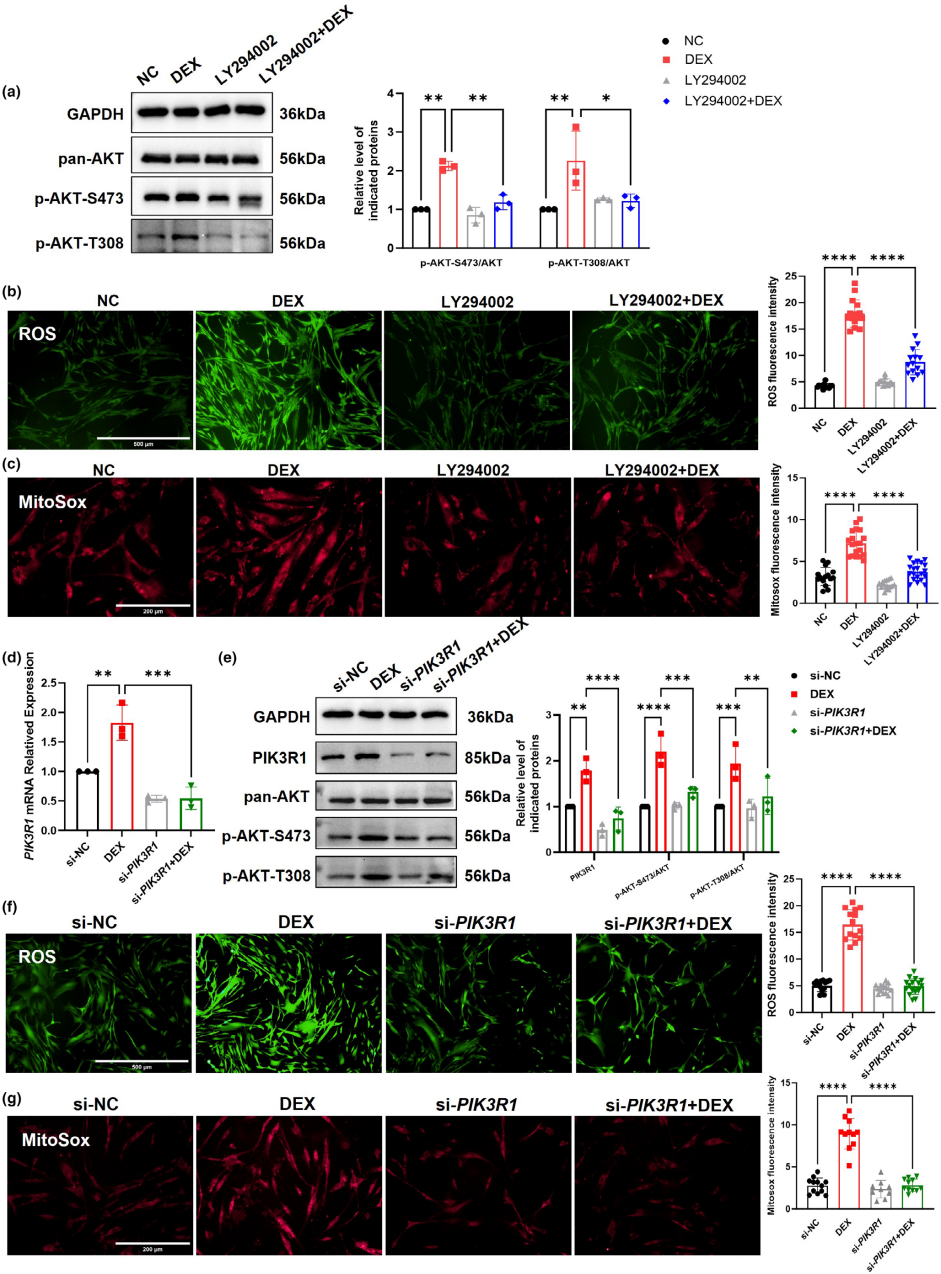
PI3K/AKT pathway inhibition alleviated DEX‐induced oxidative stress in pHTMs. (a) LY294002 (50 μM, 72 h) inhibited DEX‐induced PI3K/AKT pathway activation in pHTMs. (b) ROS levels (*n* ≥ 13 fields per group). Scale bar = 500 μm. (c) Mitochondrial superoxide levels (*n* ≥ 15 fields per group). Scale bar = 200 μm. (d) *PIK3R1* mRNA expression levels. (e) Protein levels of PIK3R1 and phosphorylation of AKT. (f) Representative ROS staining images (*n* ≥ 14 fields per group). Scale bar = 500 μm. (g) Representative mitochondrial superoxide staining images (*n* ≥ 9 fields per group). Scale bar = 200 μm. The experiments were conducted using cell strains cultured from three separate donors. Data are presented as mean ± SD. One‐way ANOVA followed by Tukey's test. *p < 0.05, **p < 0.01, ***p < 0.001, ****p < 0.0001.

To further delve into the role of PI3K/AKT pathway in GIG, PI3K inhibitor LY294002 (50 μM, 72 h) (Figure 2a) and GIG cell model were used in this study (Deng et al., 2024; Zeng et al., 2020). The results revealed a significant elevation in intracellular ROS and mitochondrial superoxide levels in the DEX‐treated group compared with the control group (Figure S4a,b). Furthermore, JC‐1 and tetramethylrhodamine ethyl ester perchlorate (TMRE) staining showed mitochondrial membrane depolarization in DEX‐treated pHTMs (Figure S4c,d), indicating damaged mitochondrial function. Inhibition of the PI3K/AKT pathway prevented DEX‐induced increases in intracellular ROS and mitochondrial superoxide levels (Figure 2b,c). Furthermore, LY294002 also protected mitochondrial function by preventing the decrease in mitochondrial membrane potential (Ψm) induced by DEX (Figure S5a,b).

To investigate the role of PIK3R1 in GIG, we employed siRNA to knock down *PIK3R1* expression in pHTMs (Figure 2d,e). *si‐PIK3R1* effectively suppressed the phosphorylation of AKT (Figure 2e), reducing cellular ROS and mitochondrial superoxide levels (Figure 2f,g) also protecting mitochondrial function by preventing the decrease in Ψm induced by DEX treatment (Figure S4c,d).

In summary, inhibition of the PI3K/AKT pathway and knockdown of *PIK3R1* inhibited DEX‐induced elevated mitochondrial superoxide levels, decreased mitochondrial Ψm, and oxidative stress in pHTMs, suggesting that PIK3R1 and PI3K/AKT pathways play a key role in DEX‐induced oxidative stress in GIG.

### 3.4 MAOA plays an important role in GIG progression and TM damage in GIG mouse models

**FIGURE 3:**
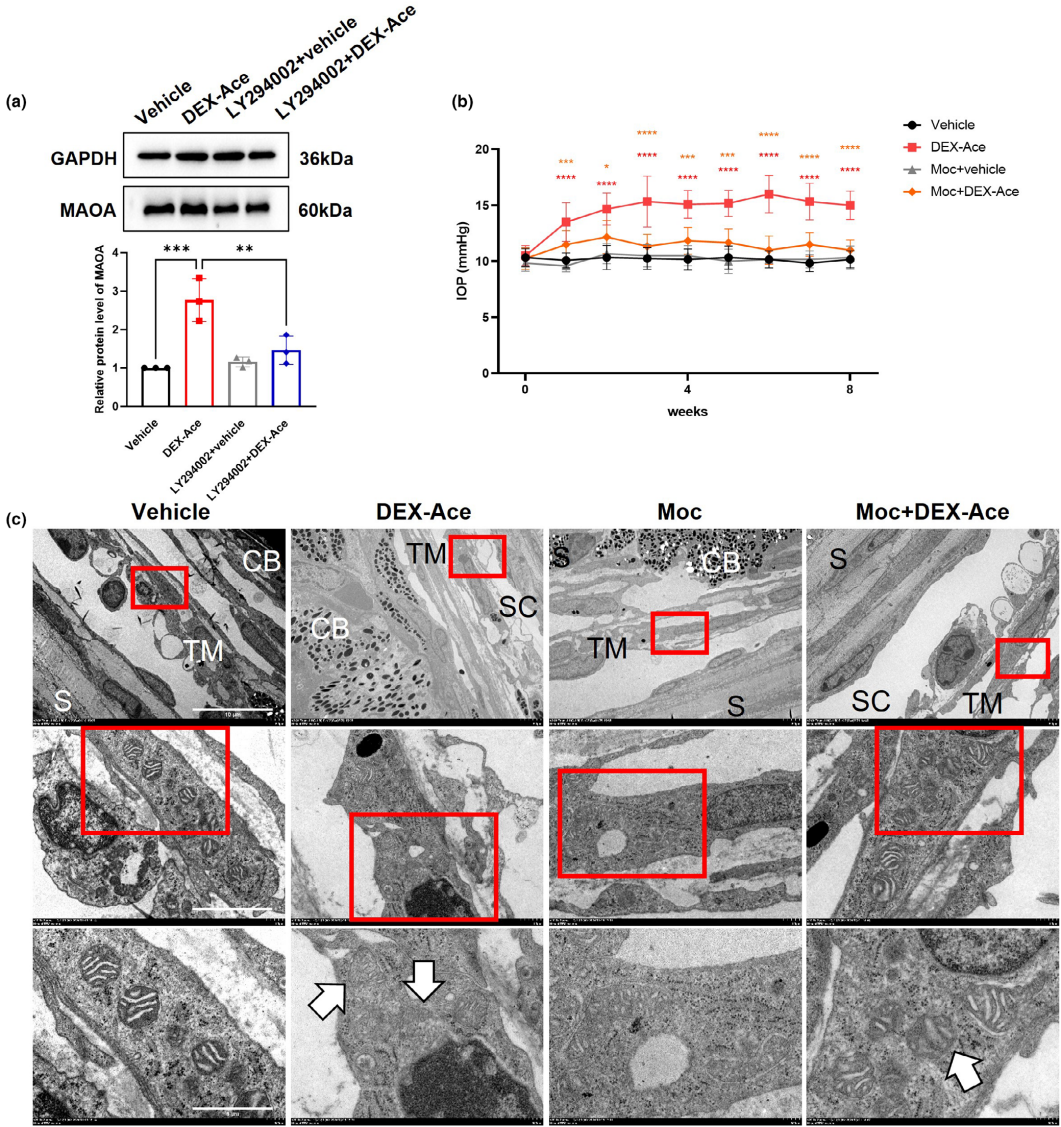
Effect of inhibiting MAOA on GIG progression in the GIG mouse model. (a) Protein expression levels of MAOA in TM of vehicle, DEX‐Ace, LY294002 + vehicle, and LY294002 + DEX‐Ace groups (*n* = 3 mouse eyes). (b) Effect of Moclobemide (Moc; MAOA inhibitor; 40 mg/kg, once every 2 days for 8 weeks) on IOP elevation in the GIG mouse model (red asterisk: Vehicle vs. DEX‐Ace; orange asterisk: DEX‐Ace vs. Moc + DEX‐Ace; *n* = 10 mouse eyes). (c) Representative images of mitochondrial morphology in the TM region from each group captured by electron microscopy. White arrows: Damaged mitochondria (n = 3 mouse eyes). Scale bar = 10 μm (upper images)/2 μm (upper images)/1 μm (lower images). CB, ciliary body; SC, Schlemm's canal; S, sclera; TM, trabecular meshwork. Data are presented as mean ± SD. One‐way ANOVA followed by Tukey's test. *p < 0.05, **p < 0.01, ***p < 0.001, ****p < 0.0001. All experiments were biologically replicated at least three times.

The precise mechanisms underlying the role of PI3K/AKT pathway on oxidative stress and GIG progression remain incompletely understood. Previous reports have shown a strong correlation between MAOA expression and ROS production. MAOA, a mitochondrial enzyme, degrades monoamine neurotransmitters and dietary amines, generating hydrogen peroxide as a primary source of ROS (Chen & Wu, 2023; Li et al., 2020). Overexpression of MAOA leads to excessive ROS production, leading to respiratory dysfunction and loss of Ψm (Maggiorani et al., 2017; Mialet‐Perez & Parini, 2020). In this study, compared with the vehicle group, DEX‐Ace treatment significantly increased the expression of MAOA (Figure 3a) in TM. WB results showed that inhibition of PI3K/AKT pathway decreased the overexpression of MAOA (Figure 3a), suggesting that the PI3K/AKT pathway may regulate MAOA to affect GIG progression.

To verify the role of MAOA in GIG, MAOA inhibitor moclobemide (Moc; an MAOA inhibitor; 40 mg/kg, once every 2 days for 8 weeks) was intraperitoneally injected into mice at the same time as establishing the GIG mouse model. Moc treatment partially prevented IOP elevation in GIG mice compared to the DEX‐Ace‐only group (Figure 3b). In addition, TEM results demonstrated that Moc treatment could attenuate mitochondrial fragmentation and damage induced by DEX‐Ace in the GIG mouse model (Figure 3c).

### 3.5 PI3K/AKT pathway modulates mitochondrial and cellular oxidative stress through regulation of MAOA in GIG

**FIGURE 4:**
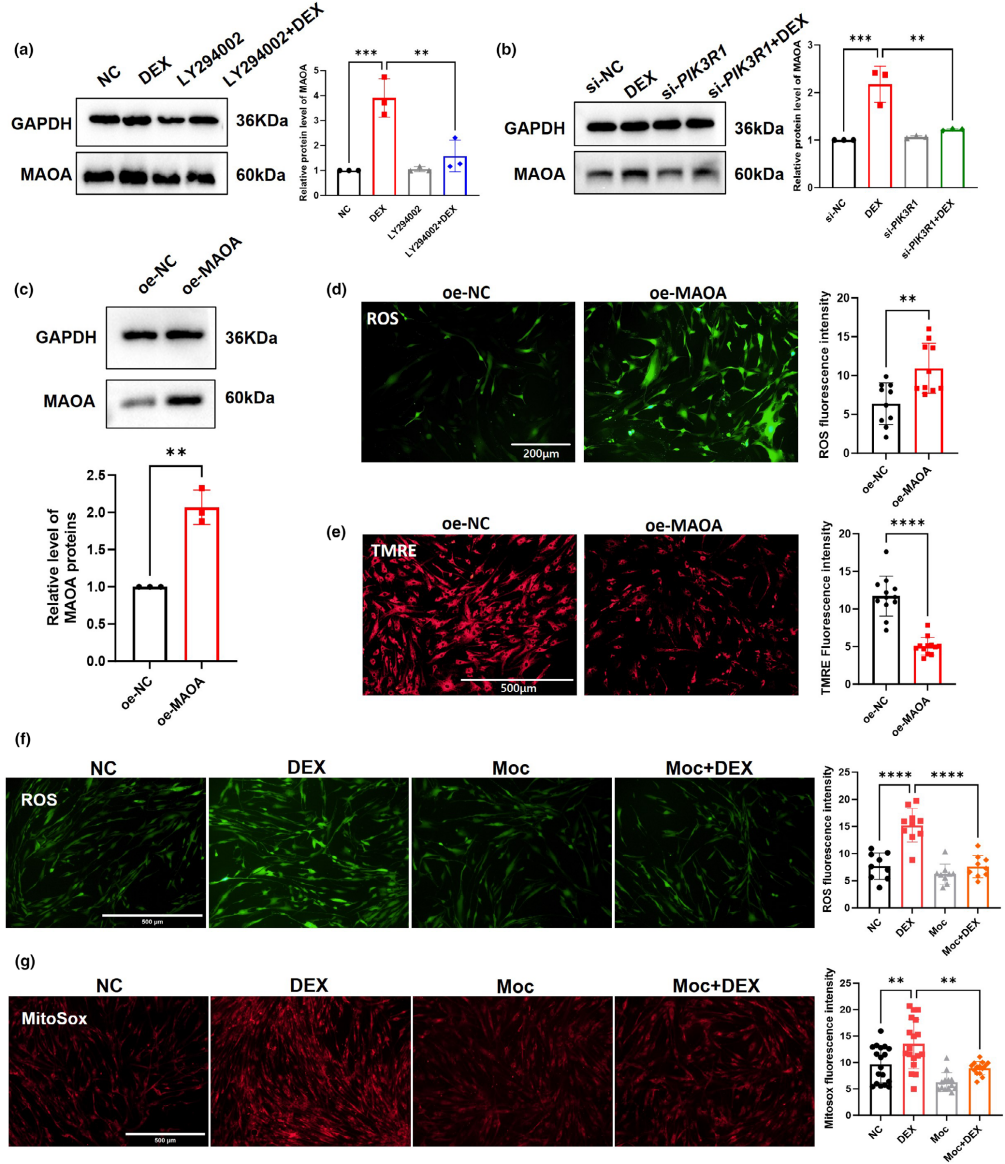
PI3K/AKT pathway regulates mitochondrial membrane enzyme MAOA, impacting pHTMs oxidative stress. (a) The effect of PI3K inhibition on MAOA overexpression in pHTMs. (b) The effect of *PIK3R1* downregulation on MAOA expression in pHTMs. (c) Expression of MAOA protein in pHTMs after overexpression of MAOA by transient transfection of plasmids. (d) Representative ROS staining images (*n* = 10 fields per group). Scale bar = 200 μm. (e) Mitochondrial membrane potential by TMRE staining (*n* = 12 fields per group). Scale bar = 500 μm. (f) Representative ROS images after Moclobemide (50 μM, 72 h) processing of pHTMs (*n* ≥ 9 fields per group). Scale bar = 500 μm. (g) Mitochondrial superoxide levels detected using MitoSOX Red (*n* ≥ 13 fields per group). Scale bar = 500 μm. The experiments were conducted using cell strains cultured from three separate donors. Data are presented as mean ± SD. Unpaired t‐test (c, d, e). One‐way ANOVA followed by Tukey's test (a, b, f, g). **p < 0.01, ***p < 0.001, ****p < 0.0001.

MAOA overexpression was also observed in pHTMs treated with DEX, and inhibition of the PI3K/AKT pathway or silencing of *PIK3R1* could suppress this overexpression (Figure 4a,b). To further confirm the role of MAOA, transient transfection of plasmids was performed to overexpress MAOA in pHTMs (Figure 4c). Compared to the oe‐NC group, overexpressing MAOA in pHTMs increased intracellular ROS levels (Figure 4d) and affected mitochondrial function (reducing Ψm), suggesting that overexpression of MAOA and DEX treatment exert similar effects on pHTMs (Figure 4e).

MAOA inhibitor moclobemide (Moc; 50 μM, 72 h) was used to inhibit MAOA and subsequently monitored oxidative stress and mitochondrial function in pHTMs after DEX treatment. Intriguingly, Moc‐mediated inhibition of MAOA decreased the levels of intracellular ROS (Figure 4f) and improved mitochondrial function (Figure 4g). Furthermore, Moc treatment did not affect the phosphorylation of AKT (Figure S6), suggesting that MAOA is downstream of the PI3K/AKT pathway. These results indicate that DEX activated the PI3K/AKT pathway, upregulating MAOA and damaging mitochondria, thereby inducing oxidative stress in GIG.

### 3.6 PI3K/AKT/MAOA pathway inhibition ameliorated premature senescence of TM in GIG

**FIGURE 5:**
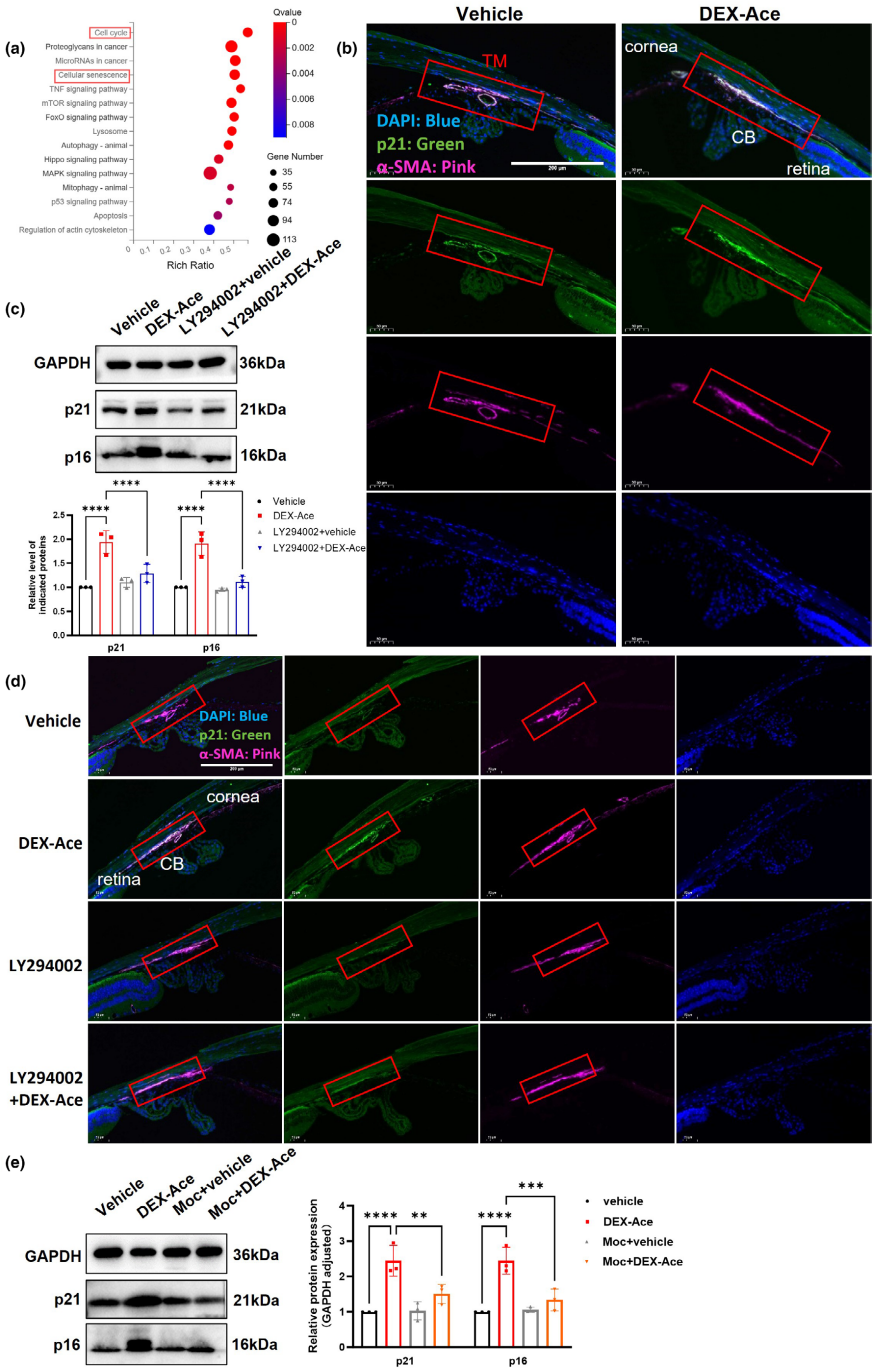
PI3K/AKT/MAOA pathway modulates TM aging in the GIG mouse model. (a) KEGG analysis of DEGs between the DEX and si‐PIK3R1 + DEX groups. (b) Representative immunofluorescence images of anterior chamber angle from the vehicle and the DEX‐Ace group after 8 weeks of DEX‐Ace treatment, p21 was stained as a senescence marker, TM region was labeled using α‐smooth muscle actin (α‐SMA), and the nuclei were stained with DAPI (*n* = 3 mouse eyes). Scale bar = 200 μm. (c) LY294002 (PI3K inhibitor; 50 mg/kg, once weekly for 8 weeks) inhibited the protein expression levels of aging markers p16 and p21 in TM of GIG mice (*n* = 3 mouse eyes). (d) Representative immunofluorescence images. LY294002 reversed the DEX‐Ace‐induced increase of p21 expression in TM. The TM region was labeled using α‐SMA, and the nuclei were stained with DAPI. Scale bar = 200 μm. (e) Moclobemide (Moc; MAOA inhibitor; 40 mg/kg, once every 2 days for 8 weeks) inhibited the protein expression levels of aging markers p16 and p21 in TM of GIG mice (*n* = 3 mouse eyes). CB, Ciliary body. Data are presented as mean ± SD. One‐way ANOVA followed by the Tukey's test. **p < 0.01, ***p < 0.001, ****p < 0.0001. All experiments were biologically replicated at least three times.

Oxidative stress and mitochondrial dysfunction are closely related to premature aging (De Gaetano et al., 2021; Korovesis et al., 2023). Reports have emphasized the crucial regulatory role of the PI3K/AKT pathway in aging phenotypes (Liu et al., 2023; Noh et al., 2016; O' NC, 2013). Significant differences in signaling pathways related to cellular senescence and cell cycle also were observed in the KEGG analysis of RNA‐sequencing data between the NC and DEX groups (Figure S3a) and also between si*PIK3R1* + DEX and DEX groups (Figure 5a). To further confirm the role of TM aging in GIG, the deposition of senescence marker p21 in the TM region of GIG mice was observed by immunofluorescence staining (Figure 5b). In GIG mice, there is an increase in p21 expression in the TM region, suggesting TM aging after DEX‐Ace treatment. Inhibition of the PI3K/AKT/MAOA pathway reduced the p21 deposition in the TM region in GIG mice and also reduced the protein expression of senescence markers p16 and p21 (Ueda et al., 2021) in TM (Figure 5c–e).

### 3.7 PI3K/AKT/MAOA pathway inhibition alleviated premature aging of pHTMs induced by DEX

To further confirm the regulation of the PI3K/AKT/MAOA pathway in aging in pHTMs. β‐galactosidase activity and cell cycle arrest, which are widely utilized as biomarkers for cellular senescence, were used to assess cellular senescence (Kumari & Jat, 2021; Lee et al., 2006). Compared to the control group, the DEX‐treated group exhibited a significant increase in both β‐galactosidase activity and the proportion of cells arrested in the G0‐G1 phase (Figure S7a,b). Furthermore, the expression of p16 and p21 was upregulated in pHTMs after DEX treatment (Figure S7c).

**FIGURE 6:**
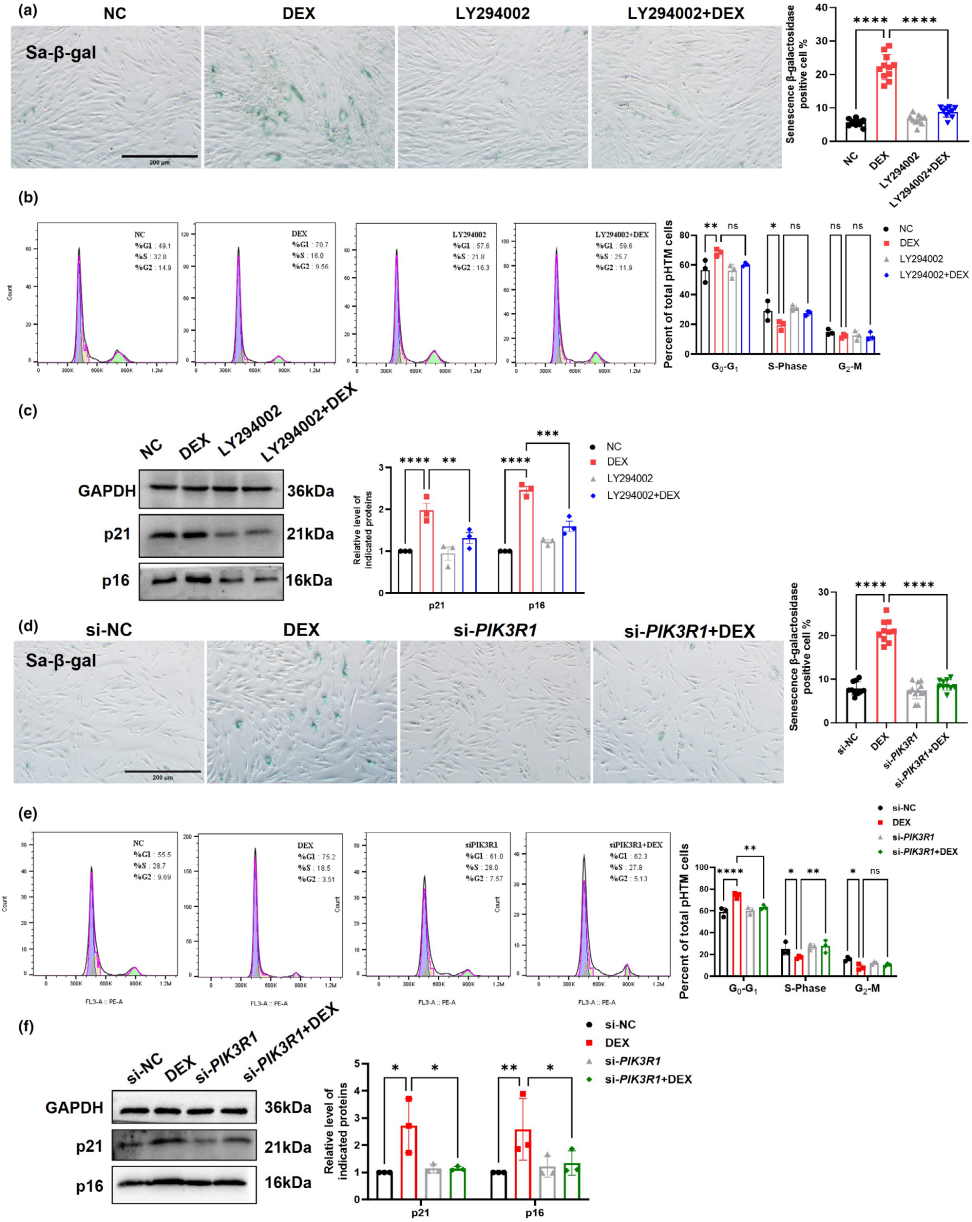
PI3K/AKT pathway inhibition prevented DEX‐induced pHTMs premature senescence. (a) Percentage of senescent cells assessed using Sa‐β‐galactosidase staining after LY294002 (50 μM, 72 h) treatment (*n* ≥ 10 fields per group). Scale bar = 200 μm. (b) Cell cycle changes detected by flow cytometry with PI staining. (c) Expression of p21 and p16 at the protein level. (d) Percentage of senescent cells assessed using Sa‐β‐galactosidase staining after knocking down *PIK3R1* (*n* ≥ 10 fields per group). Scale bar = 200 μm. (e) Cell cycle changes detected by flow cytometry with PI staining. (f) Expression of p21 and p16 at the protein level. The experiments were conducted using cell strains cultured from three separate donors. Data are presented as mean ± SD. One‐way ANOVA followed by Tukey's test. *p < 0.05, **p < 0.01, ****p < 0.0001.

The proportion of Sal‐β‐gal‐positive cells in DEX‐treated group was reduced after treatment with LY294002 (50 μM, 72 h) (Figure 6a). Treatment with LY294002 also prevented DEX‐induced cell cycle arrest and reduced the protein levels of senescence markers p16 and p21 (Figure 6b,c). Silencing PIK3R1 in pHTMs produces the same effect as PI3K/AKT pathway inhibition (Figure 6d–f).

**FIGURE 7:**
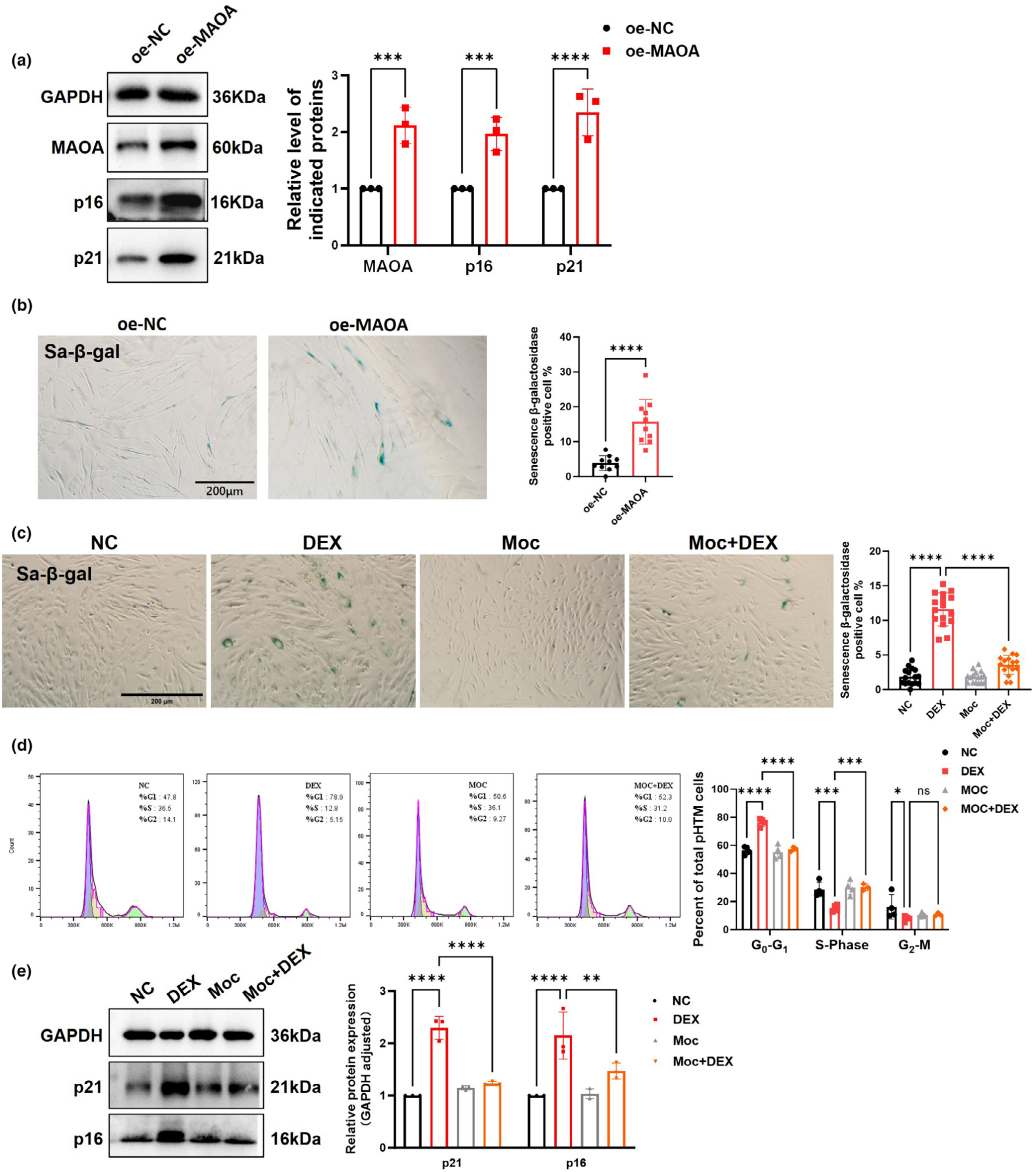
Inhibition of MAOA attenuates DEX‐induced premature senescence in pHTMs. (a) MAOA overexpression increased the protein expression of p21 and p16. (b) The percentage of β‐galactosidase positive cells quantified using Sa‐β‐gal staining (*n* ≥ 10 fields per group). Scale bar = 200 μm. (c) Representative images for Sa‐β‐gal staining after Moclobemide (50 μM, 72 h) treatment (*n* = 16 fields per group). Scale bar = 200 μm. (d) Cell cycle changes of pHTMs detected by flow cytometry with PI staining. (e) Effect of Moc‐induced inhibition of MAOA on p21 and p16 levels in DEX‐treated pHTMs. The experiments were conducted using cell strains cultured from three separate donors. Data are presented as mean ± SD. Unpaired t‐test (a, b). One‐way ANOVA followed by the Tukey's test (c, d, e). *p < 0.05, **p < 0.01, ***p < 0.001, ****p < 0.0001.

Overexpression of MAOA in pHTMs induced increased expression of p21 and p16 (Figure 7a), also elevated ratio of Sal‐β‐gal‐positive cells (Figure 7b) compared with oe‐NC group. In turn, the treatment of Moc (MAOA inhibitor; 50 μM, 72 h) significantly inhibited premature pHTMs senescence, with a reduced ratio of Sal‐β‐gal‐positive cells, inhibited cell cycle arrest and downregulation of p21 and p16 (Figure 7c–e).

In summary, these findings support our hypothesis that DEX activates the PI3K/AKT/MAOA pathway in TM, leading to increased mitochondrial superoxide production, mitochondrial dysfunction, and ultimately oxidative stress and premature aging in TM, which results in GIG progression.

## 4 DISCUSSION

IOP elevation in glaucoma is intimately linked to TM dysfunction, prompting numerous GIG studies to focus on the roles of TM cells (Zode et al., 2014). The aging of TM cells is a major risk factor for the development or progression of glaucoma and GIG pathology (Chhunchha et al., 2017; Du et al., 2022). Additionally, DEX triggers mitochondrial dysfunction, increases ROS levels, and induces oxidative stress and senescence‐related alterations in TM cells (He et al., 2019; Zeng et al., 2020). Oxidative stress is an important factor promoting premature cell aging (Faraonio, 2022; Wiley & Campisi, 2021).

In this study, GCs treatment accelerated oxidative stress‐induced TM senescence in vivo and in vitro. We applied high‐throughput mRNA sequencing, highlighting the PI3K/AKT pathway as pivotal in TM senescence. Integrating our findings with GEO data, we identified PIK3R1 as a key molecule. Further exploration of anti‐ROS/anti‐aging therapies for TM preservation in glaucoma is necessary.

*PIK3R1 en*codes the main class I PI3K regulator, with the PI3K/AKT pathway crucial for cell functions like proliferation, metabolism, survival, senescence, and migration (Karar & Maity, 2011; Porta et al., 2014). Silencing *PIK3R1* or blocking PI3K/AKT decreases ROS, thereby reducing oxidative stress in DEX‐treated pHTMs, which emphasizes PI3K/AKT's role in TM premature senescence. Moreover, DEX activates PI3K/AKT to regulate MAOA, with MAOA overexpression mirroring GC damage in TM, underlining its significance in GIG.

Oxidative stress results from an imbalance between intracellular oxidation and antioxidation processes, leading to a pro‐oxidative state (Sies, 2020). This state is due to increased levels of oxidants such as ROS and reactive nitrogen species, causing damage to cells and tissues. Chronic oxidative stress is a significant factor in cellular senescence and the development of various diseases (Lu et al., 2023).

MAOA, a mitochondrial enzyme, catalyzes the oxidative deamination of monoamine neurotransmitters and dietary amines, requiring flavin adenine dinucleotide covalently bound to it as a cofactor (Di Sante et al., 2023). This process produces hydrogen peroxide as a byproduct, which is a major source of ROS (Wang et al., 2021). Therefore, overexpression of MAOA significantly affects mitochondrial status and function. Inhibiting MAOA with Moc can protect mitochondrial function, reduce elevated ROS levels, and rescue DEX‐induced senescence changes, suggesting that activated MAOA plays a crucial role in GIG.

Collectively, our results demonstrate that DEX induces overexpression of PI3KR1 at both RNA and protein levels, activating the PI3K/AKT pathway and leading to increased MAOA expression, inducing cellular oxidative stress and premature aging. While this study has yielded significant findings, unresolved issues necessitate further investigation. Understanding the impact of PI3K/AKT pathway on MAOA and the interplay between them is imperative. Furthermore, exploring the supplementary mechanisms by which DEX and other GCs contribute to TM degeneration is essential for a more thorough understanding of this multifaceted process. This research provides novel insights into potential therapeutic strategies for addressing GIG and oxidative stress‐induced cellular senescence in glaucoma.

## 5 CONCLUSION

This study evaluates the role of PI3K/AKT/MAOA in premature TM aging in GIG. Our findings indicate that DEX treatment increases oxidative stress in pHTMs, promoting cellular senescence. Mechanistically, DEX activates the PI3K/AKT/MAOA pathway in TM, affecting mitochondrial function, resulting in increased production of ROS, inducing premature aging of TM and promoting the occurrence and development of GIG. This provides new possibilities for the prevention and treatment of TM senescence and glaucoma.

## AUTHOR CONTRIBUTIONS

**Pengyu Zhang**: Methodology, investigation, formal analysis, writing—original draft; data curation. **Nan Zhang**: Investigation, formal analysis, writing—original draft, data curation. **Yixin Hu**: Validation, supervision. **Xizhi Deng**: Investigation. **Min Zhu**: Supervision and review and editing. **Cheng Lai**: Investigation. **Wen Zeng**: Review and editing. **Min Ke** (Responding author): Methodology, validation, supervision, resources, review and editing. All authors made substantial contributions to the conception and design of the article, drafting or revising the article for critically important intellectual content, and approved the final version of the article.

## FUNDING INFORMATION

This research was supported by the Hubei Provincial Natural Science Foundation of China (Grant No. 2023AFB053), China Postdoctoral Science Foundation (Grant No. 2022M722463), and National Natural Science Foundation of China (Grant No. 82271088).

## CONFLICT OF INTEREST STATEMENT

The authors declare that they do not have financial or other conflicts of interest.

## DISCLOSURES

The authors declare no commercial relationships.

## Supporting information


Data S1:

## Data Availability

A portion of the RNA‐seq data relevant to this study was obtained from publicly available sources in the GEO database (https://www.ncbi.nlm.nih.gov/geo/; GSE16643, GSE37474, and GSE124114). The RNA‐seq data that were independently measured by the researchers can be accessed upon submitting a reasonable request to the corresponding author. Apart from the RNA‐seq data, all other data pertinent to this research have been presented within the main text of the paper or its supplementary materials.
