# Supplementary material for: Role of PI3K/AKT/MAOA in glucocorticoid‐induced oxidative stress and associated premature senescence of the trabecular meshwork
Source: Aging Cell. 2024 Dec 17;24(4):e14452. doi: 10.1111/acel.14452 (PMC11984687; doi:10.1111/acel.14452)
Supplement: Supplementary file 1 — Data S1: [file ACEL-24-e14452-s001.zip › revised clean copy-Supplementary materials 1-supplementary methods and figures.pdf]

1. Supplementary Methods

1.1 Immunofluorescence

pHTMs were fixed, permeabilized, and blocked for 2 hours in a blocking buffer containing 1% BSA. Subsequently, they were incubated with primary antibodies (1:200, MGP, Myocilin) at 4 °C overnight and secondary antibodies (1:200; goat anti-rabbit Alexa Fluor 488 [GB22403, Servicebio], and anti-rabbit Alexa Fluor 594 [GB21403, Servicebio]) for 2 hours at room temperature. Nuclei were stained with 4′,6-diamidino-2-phenylindole (DAPI; Sigma, D9542). Microscopy (Olympus, Japan) and ImageJ (version 1.52) software were used for image acquisition and analysis, respectively.

1.2 Immunohistochemistry

For TM tissue samples, the TM paraffin sections are first dewaxed using xylene and then rehydrated using a gradient alcohol series. After antigen retrieval with antigen retrieval solution (P0083, Beyotime, Shanghai, China), the sections are treated with 0.3% Triton X-100 for 10 minutes and washed three times with PBS. Following this, the sections are incubated in a 3% BSA solution at room temperature for 2 hours and then with the primary antibody (1:200, p21 [A19094, ABclonal Technology, China], α-SMA [395-1-AP, Proteintech, Wuhan, China]) overnight at 4°C. After five washes with PBS, the sections are incubated with the secondary antibody for 2 hours, followed by another five washes. Finally, the sections are mounted using an antifade mounting medium containing DAPI (P0131, BioTium, Shanghai, China), which stains the nuclei. Images are captured under a fluorescence microscope (IX71, Olympus, Tokyo, Japan).

1.3 RNA seq

Total RNA from different treatment groups of pHTMs was extracted using TRIzol reagent (Invitrogen, Thermo Fisher Scientific, MA, USA) following the manufacturer's instructions. cDNA libraries were loaded onto a BGIseq500 platform (BGI-Shenzhen, Shenzhen, China) using the Agilent 2100 bioanalyzer (Thermo Fisher Scientific, MA, USA). Sequencing was performed in triplicate for each group. Raw sequencing data

were filtered to obtain clean data using SOAPnuke (v1.5.6) and were further analyzed and mapped using Dr. Tom's multi-omics data-mining system (https://biosys.bgi.com). Gene expression quantification was conducted using RSEM (v1.3.1). The results were visualized using pheatmap (v1.0.8). The detection of differentially expressed genes (DEGs) was performed using DESeq2 (v1.4.5) (|log fold change [FC]| ≥1, adjusted $P<0.05$). Gene Ontology (GO) and Kyoto Encyclopedia of Genes and Genomes (KEGG) enrichment analyses were carried out using Phyper (https://en.wikipedia.org/wiki/Hypergeometric_distribution), with a significance threshold set at Q value ≤ 0.05.

1.4 Real-time qPCR (RT qPCR)

Total cellular RNA was isolated from control and DEX-treated groups using the HiPure Total RNA Plus Kit (MAGEN, Guangzhou, China) as per the manufacturer's instructions. HiScript III RT SuperMix for qPCR (Vazyme, Nanjing, China) was employed to reverse transcribe RNA into cDNA. Primers for *PIK3R1* (5'-AAGAAGTTGAACGAGTGGTTGG-3' and 5'-GCCCTGTTTACTGCTCTCCC-3'), *GAPDH* (5'-ACAACTTTGGTATCGTGGAAGG-3' and 5'-GCCATCACGCCACAGTTTC-3'), *Myocilin* (5'-GGCCACCAAAGCTCGACTC-3' and 5'-GAGGTTGCTGTAGGCAGTCT-3') and *p110a* (5'-CCACGACCATCATCAGGTGAA-3' and 5'-CCTCACGGAGGCATTCTAAAGT-3') were designed and manufactured by Tsingke Biotechnology (Shanghai, China). Subsequently, the CFX 96™ Real-Time PCR detection system (Bio-Rad Hercules, CA, USA) and Taq Pro Universal SYBR qPCR Master Mix (Vazyme, Nanjing, China) were used for RT-qPCR. Data analysis was conducted using the ΔΔCt method with normalization to glyceraldehyde 3-phosphate dehydrogenase (*GAPDH*).

1.5 Mitochondrial membrane potential (Ψm)

Mitochondrial Membrane Potential Detection kit (abs50016, Absin, China) and chloroquine tetrachloroiodide rhodamine B ester (TMRE, HY-D0985A, MedChemExpress) staining were used to assess Ψm. JC-1 probe and TMRE were

prepared according to the manufacturer's protocol. The JC-1 probe was added to the cells and incubated at 37 °C in the dark for 20 minutes. TMRE at a concentration of 80 nM was added to the culture medium of pHTMs and incubated in the dark at 37 °C for 15 minutes. Fluorescence was observed under a microscope (Olympus, Japan) and analyzed using ImageJ (version 1.52) software.

2. Antibody List

2.1 Primary antibodies:

rabbit anti-p16 (1:1000, A5025, ABclonal Technology, China)

rabbit anti-p21 (1:1000, A19094, ABclonal Technology, China)

rabbit anti-PIK3R1 (1:1000, A4992, ABclonal Technology, China)

rabbit anti-p110a (1:1000, A0265, ABclonal Technology, China)

rabbit anti-MAOA (1:1000, A4105, ABclonal Technology, China)

rabbit anti-pan-AKT (1:1000, #4691, Cell Signaling Technology, Boston, USA)

rabbit anti-p-AKT-S473 (1:1000, #4060, Cell Signaling Technology, Boston, USA)

rabbit anti-p-AKT-T308 (1:1000, AP1332, ABclonal Technology, China)

rabbit anti-Myocilin (1:1000, A1589, ABclonal Technology, China)

mouse anti-glyceraldehyde 3-phosphate dehydrogenase (GAPDH, 1:5000, Proteintech, 60004-1-Ig)

2.2 Secondary antibody:

goat anti-rabbit IgG secondary antibody (1:5000, AS014, ABclonal, Wuhan, China)

goat anti-mouse IgG secondary antibody (1:5000, AS003, ABclonal Technology, China)

3. Supplementary Figures

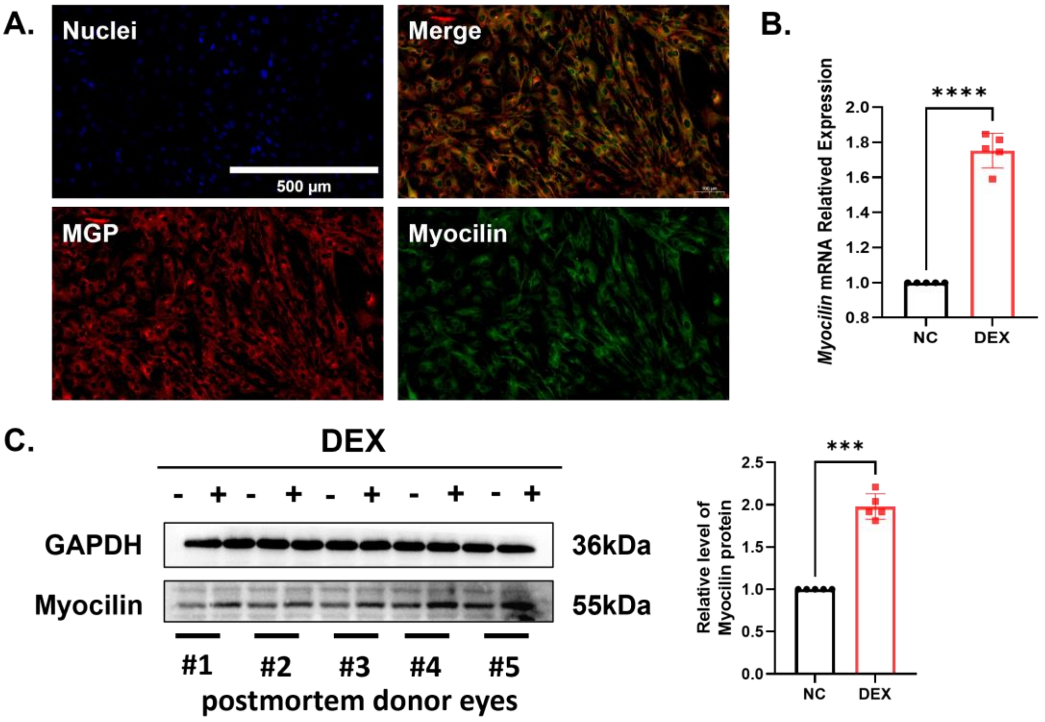

Supplementary Fig. 1. Cell identification of primary human trabecular meshwork cells.

A. Myocilin and Matrix Gla Protein (MGP) immunofluorescence staining were used for cell identification. Scale bar = 500 µm. **B.** mRNA expression level of myocilin after DEX treatment were used for pHTMs cell identification (n=5). **C.** Myocilin protein expression level after DEX treatment (n=5). All five cell lines obtained from the five postmortem donor eyes were treated with DEX and expressed and secreted myocilin protein in response to DEX. Data are presented as mean ± SD. Unpaired t-tests were utilized to assess the significance of the results. ***$P < 0.001$, ****$P < 0.0001$.

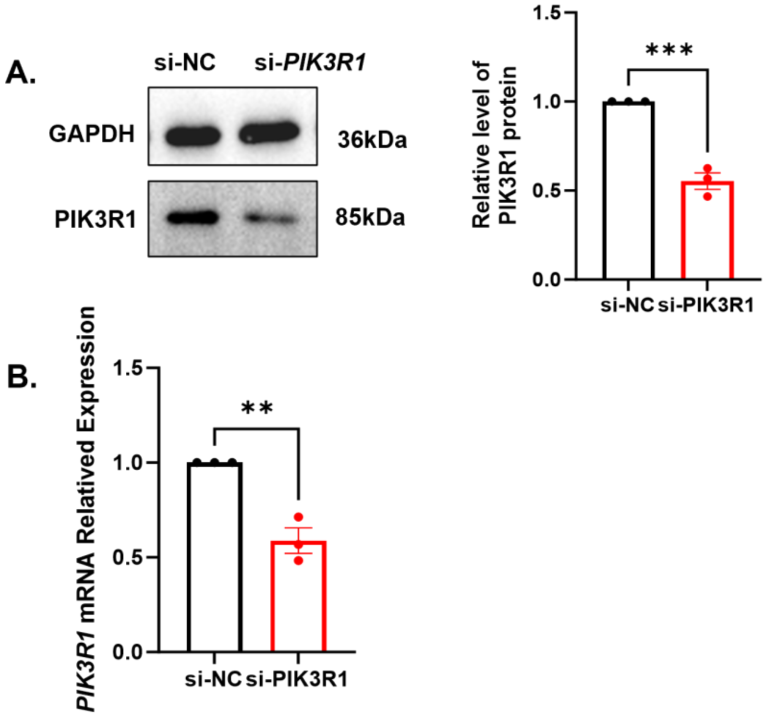

Supplementary Fig. 2. Knockdown of *PIK3R1* in pHTMs

A, B. Using RT-PCR and WB to detect the mRNA and protein expression levels of PIK3R1 in pHTMs, respectively, as indicators of siRNA knockdown efficiency. The experiments were conducted using cell strains cultured from three separate donors. Data are presented as mean ± SD. Unpaired t-tests were utilized to assess the significance of the results. $^{**}P < 0.01$, $^{***}P < 0.001$.

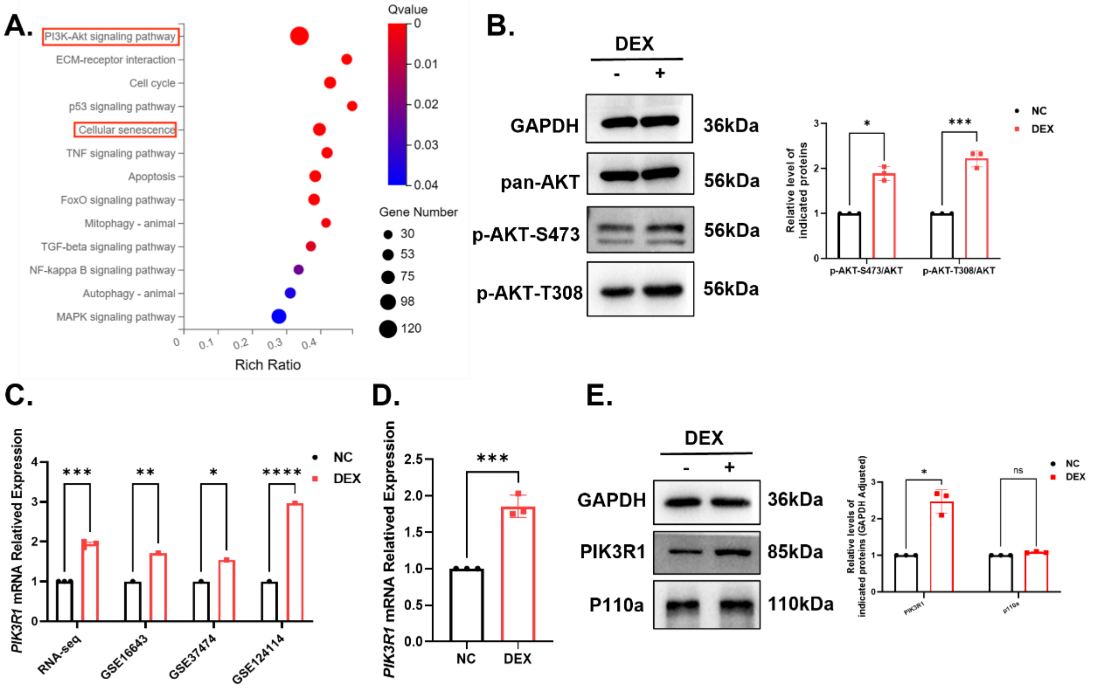

Supplementary Fig. 3. DEX induced activation of PI3K/AKT pathway.

A. KEGG analysis of DEGs between the control and DEX groups. **B.** Protein expression levels of pan-AKT, p-AKT-S473 and p-AKT-T308 in pHTMs. **C.** The relative expression levels of PIK3R1 in the control group and DEX group were shown in our RNA sequencing results, GSE16643, GSE37474, and GSE124114. **D.** Relative mRNA expression levels of PIK3R1 assessed using qPCR. **E**. Protein expression levels of p110a and PIK3R1 in pHTMs. **B&D&E** were conducted using cell strains cultured from three separate donors. Data are presented as mean ± SD. Unpaired t-tests were utilized to assess the significance of the results. *P < 0.05, **P < 0.01, ***P < 0.001, ****P < 0.0001.

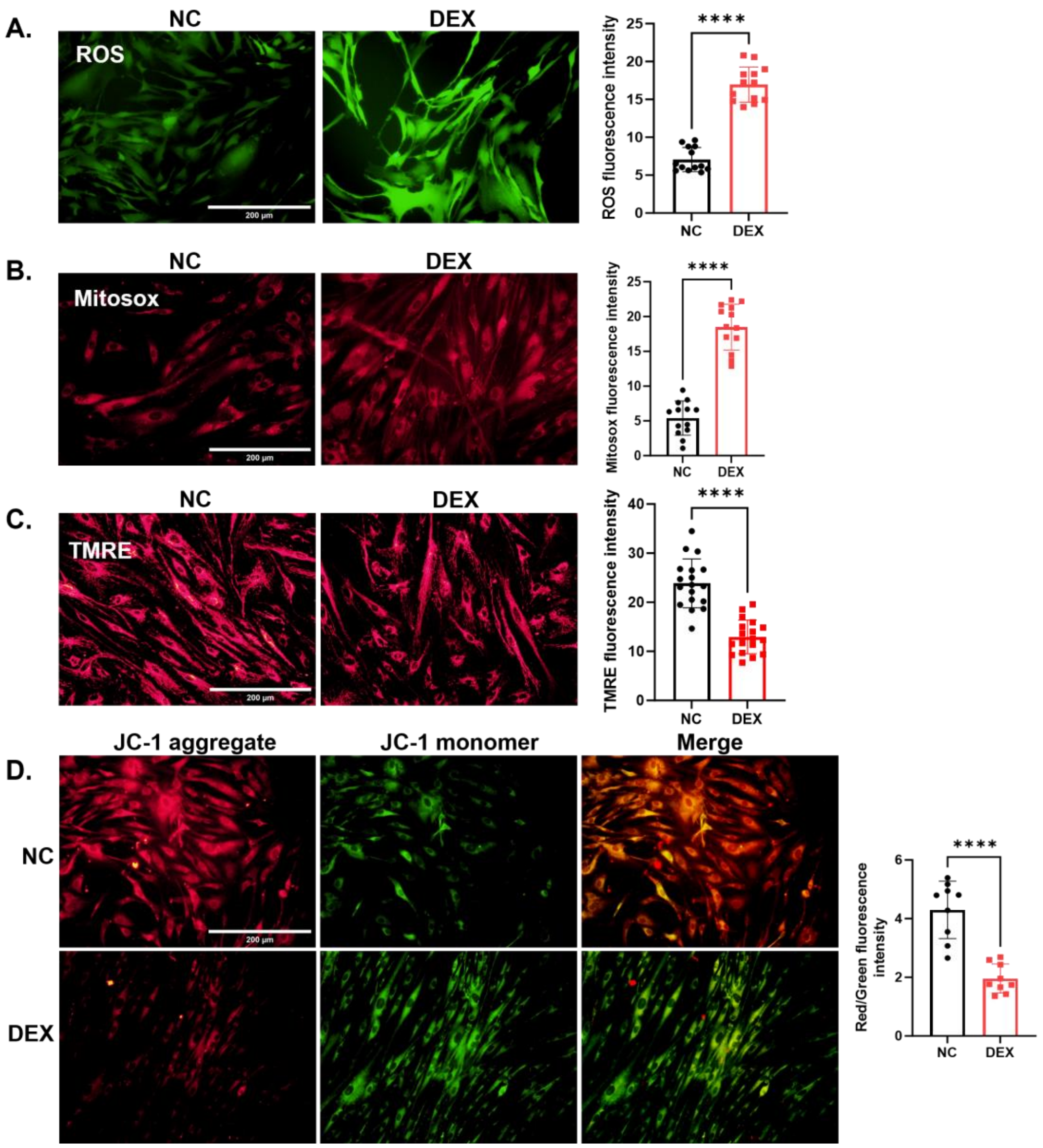

Supplementary Fig. 4. Effects of DEX on oxidative stress, mitochondrial superoxide levels and mitochondrial membrane potential in pHTMs

A. Representative ROS staining images (n = 13 fields per group). Scale bar = 200 μm. **B.** Representative mitochondrial superoxide staining images (n = 13 fields per group). Scale bar = 200 μm. Mitochondrial membrane potential assessed using TMRE **(C)** and JC-1 **(D)** staining (**C, D:** n ≥ 9 fields per group). Scale bar = 200 μm (**C&D**). The experiments were conducted using cell strains cultured from three separate donors. Data are presented as mean ± SD. Unpaired t-tests were utilized to assess the significance of the results. *P < 0.05, **P < 0.01, ***P < 0.001, ****P < 0.0001. The experiment was biologically replicated three times.

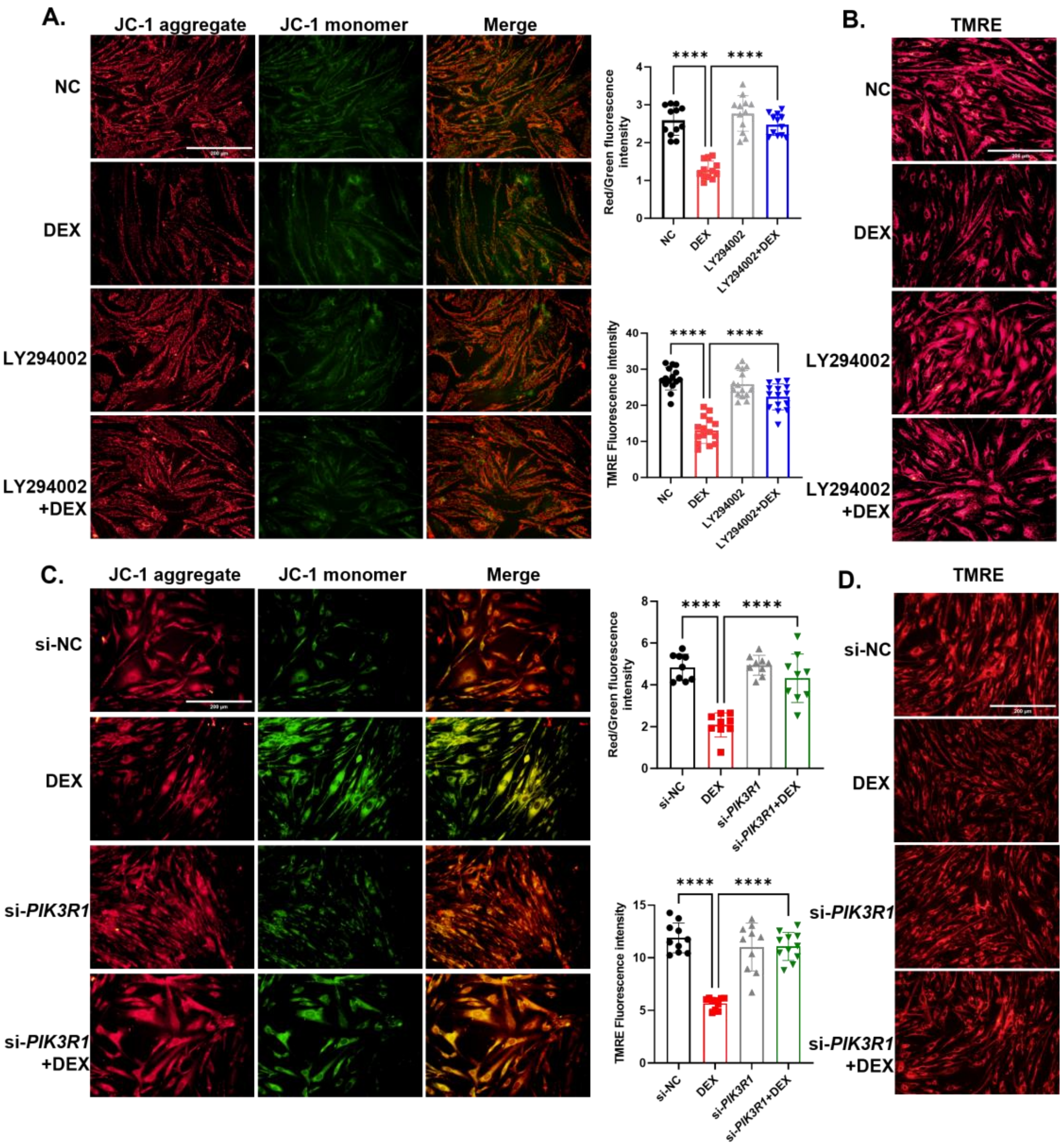

Supplementary Fig. 5. Knocking down of PIK3R1 and inhibiting PI3K in vitro prevented DEX-induced mitochondrial dysfunction in pHTMs

Effect of LY294002-induced PI3K inhibition on **A.** Mitochondrial membrane potential by JC-1. Scale bar = 200 μm. **B.** Mitochondrial membrane potential by TMRE. Scale bar = 200 μm. Effect of PIK3R1 knockdown on mitochondrial membrane potential of pHTMs assessed using JC-1 **(C)** and TMRE **(D)** staining. Scale bar = 200 μm (**A-D:** n ≥ 9 fields per group). The experiments were conducted using cell strains cultured from three separate donors. Data are presented as mean ± SD. One-way ANOVA followed

by Tukey's test. ****P < 0.0001. The experiment was biologically replicated at least three times.

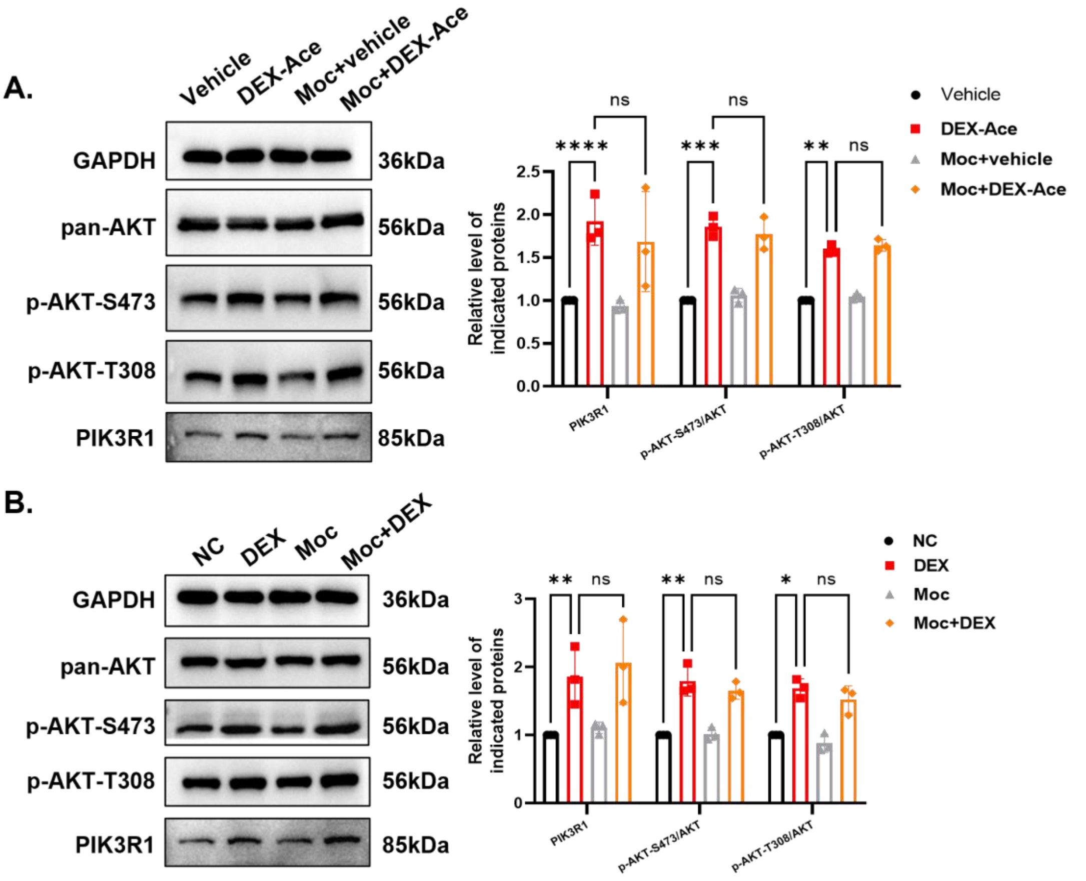

Supplementary Fig. 6. Expression of PIK3R1 and phosphorylation of AKT in TM after Moc treatment

A. PIK3R1 and phosphorylation of AKT in TM (n=3 mouse eyes). **B.** Effect of Moc-induced inhibition of MAOA on phosphorylation of PIK3R1 and AKT in DEX-treated pHTMs. The experiment was conducted using cell strains cultured from three separate donors. Data are presented as mean ± SD. One-way ANOVA followed by Tukey's test. *P < 0.05, **P < 0.01, ***P < 0.001, ****P < 0.0001.

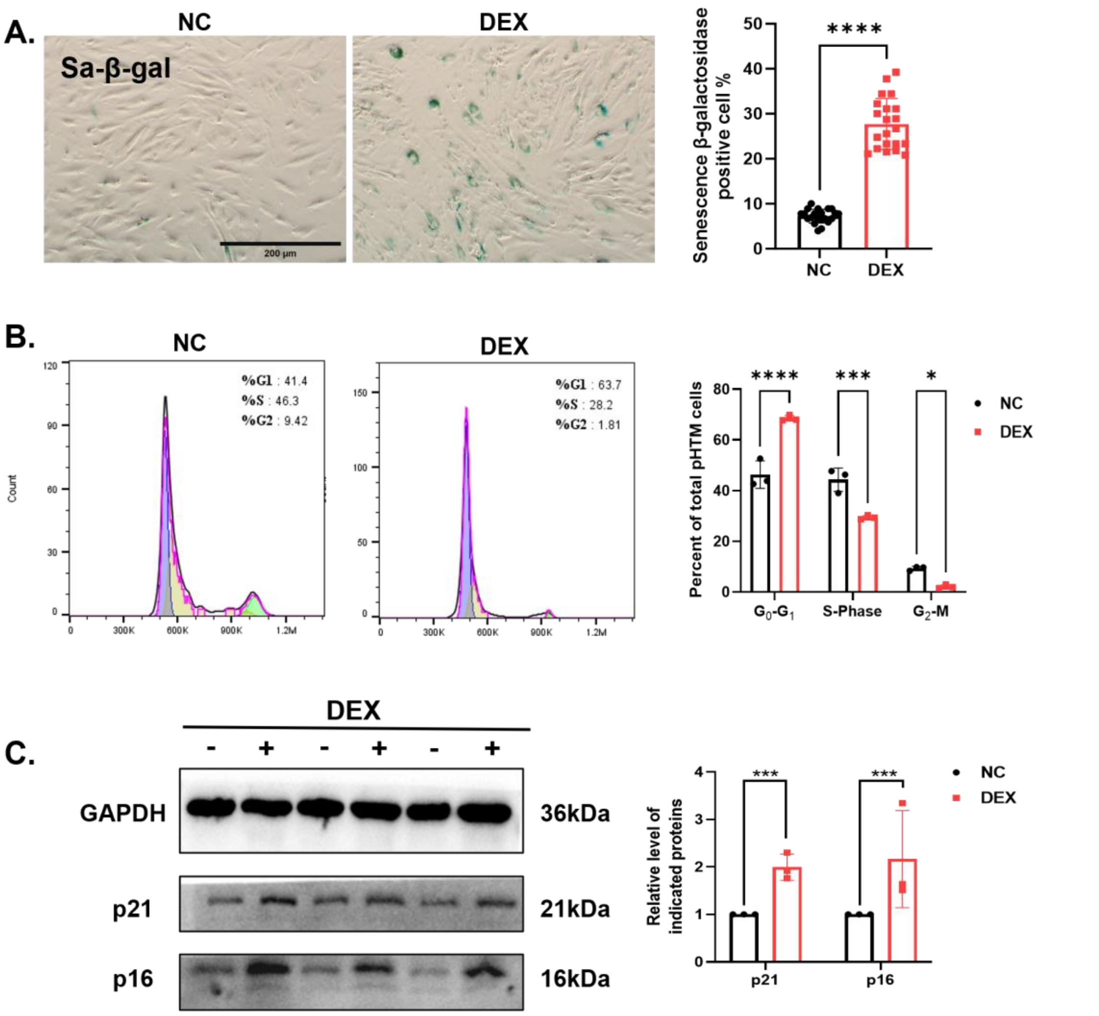

Supplementary Fig. 7. DEX induces oxidative stress and senescence phenotype in pHTMs

A. Senescent pHTMs were identified through senescence-associated β-galactosidase staining (n ≥ 15 fields per group). Scale bar = 200 μm. **B.** Cell cycle changes in DEX-treated pHTMs assessed using flow cytometry. **C.** Protein levels of aging markers p21 and p16 assessed using Western blot. The experiments were conducted using cell strains cultured from three separate donors. Data are presented as mean ± SD. Unpaired t-tests were utilized to assess the significance of the results. *P < 0.05, **P < 0.01, ***P < 0.001, ****P < 0.0001. The experiment was biologically replicated three times.
