# Supplementary material for: Role of PI3K/AKT/MAOA in glucocorticoid‐induced oxidative stress and associated premature senescence of the trabecular meshwork
Source: Aging Cell. 2024 Dec 17;24(4):e14452. doi: 10.1111/acel.14452 (PMC11984687; doi:10.1111/acel.14452)

Supplementary materials 2-Uncropped Western Blot images

1. Figure 1B

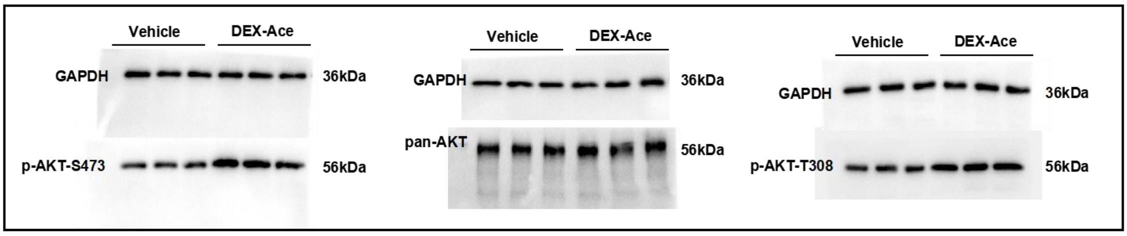

2. Figure 1C

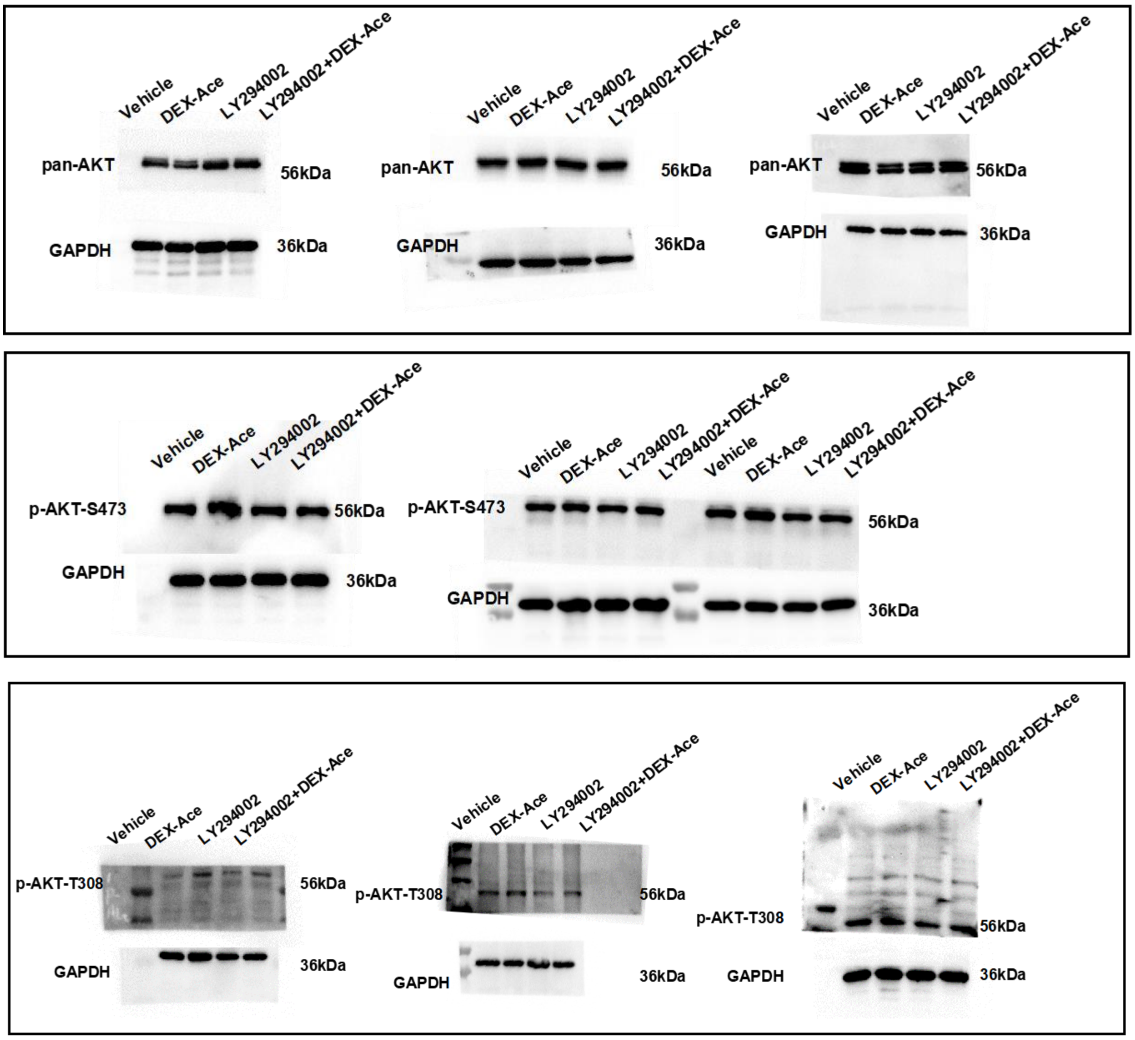

3. Figure 2A

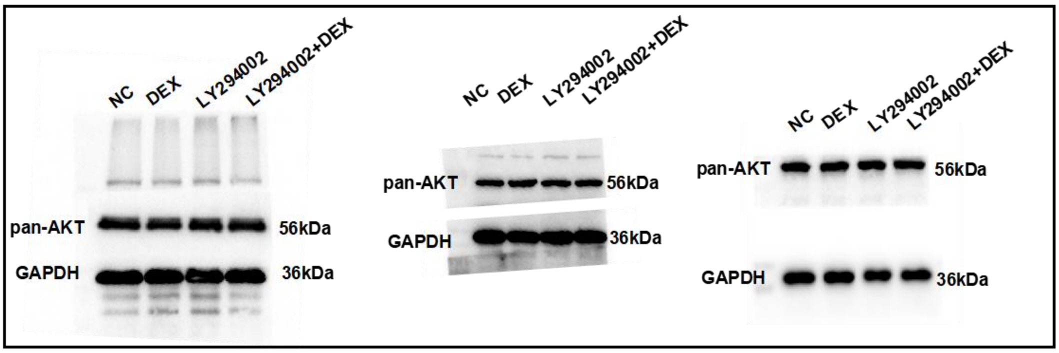

NC
DEX
LY294002
LY294002+DEX
pan-AKT
56kDa
GAPDH
36kDa
NC
DEX
LY294002
LY294002+DEX
pan-AKT
56kDa
GAPDH
36kDa
NC
DEX
LY294002
LY294002+DEX
pan-AKT
56kDa
GAPDH
36kDa

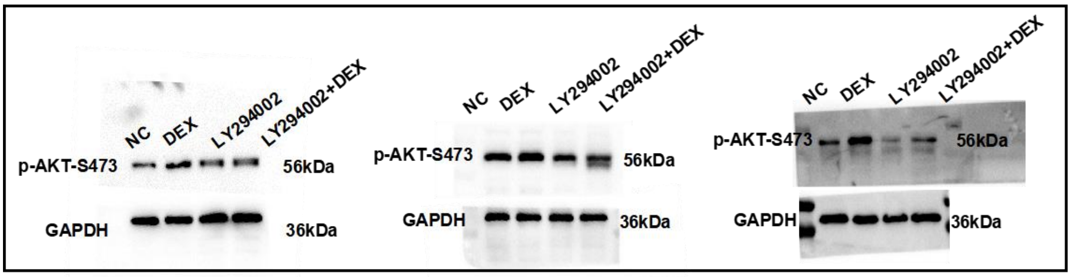

NC
DEX
LY294002
LY294002+DEX
p-AKT-S473
56kDa
GAPDH
36kDa
NC
DEX
LY294002
LY294002+DEX
p-AKT-S473
56kDa
GAPDH
36kDa
NC
DEX
LY294002
LY294002+DEX
p-AKT-S473
56kDa
GAPDH
36kDa

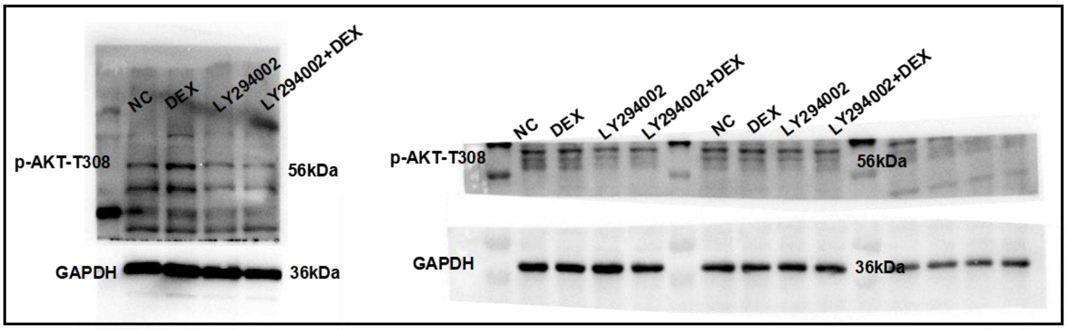

NC
DEX
LY294002
LY294002+DEX
p-AKT-T308
56kDa
GAPDH
36kDa
NC
DEX
LY294002
LY294002+DEX
NC
DEX
LY294002
LY294002+DEX
p-AKT-T308
56kDa
GAPDH
36kDa

4. Figure 2E

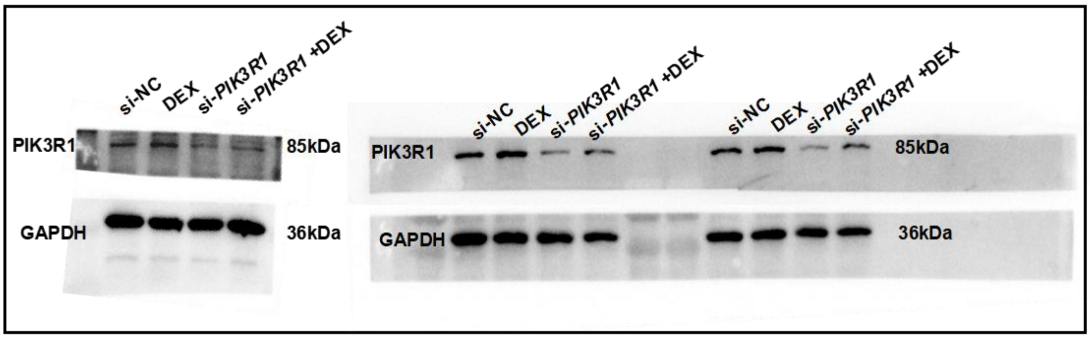

si-NC
DEX
si-PIK3R1
si-PIK3R1 +DEX
PIK3R1
85kDa
GAPDH
36kDa
si-NC
DEX
si-PIK3R1
si-PIK3R1 +DEX
si-NC
DEX
si-PIK3R1
si-PIK3R1 +DEX
PIK3R1
85kDa
GAPDH
36kDa

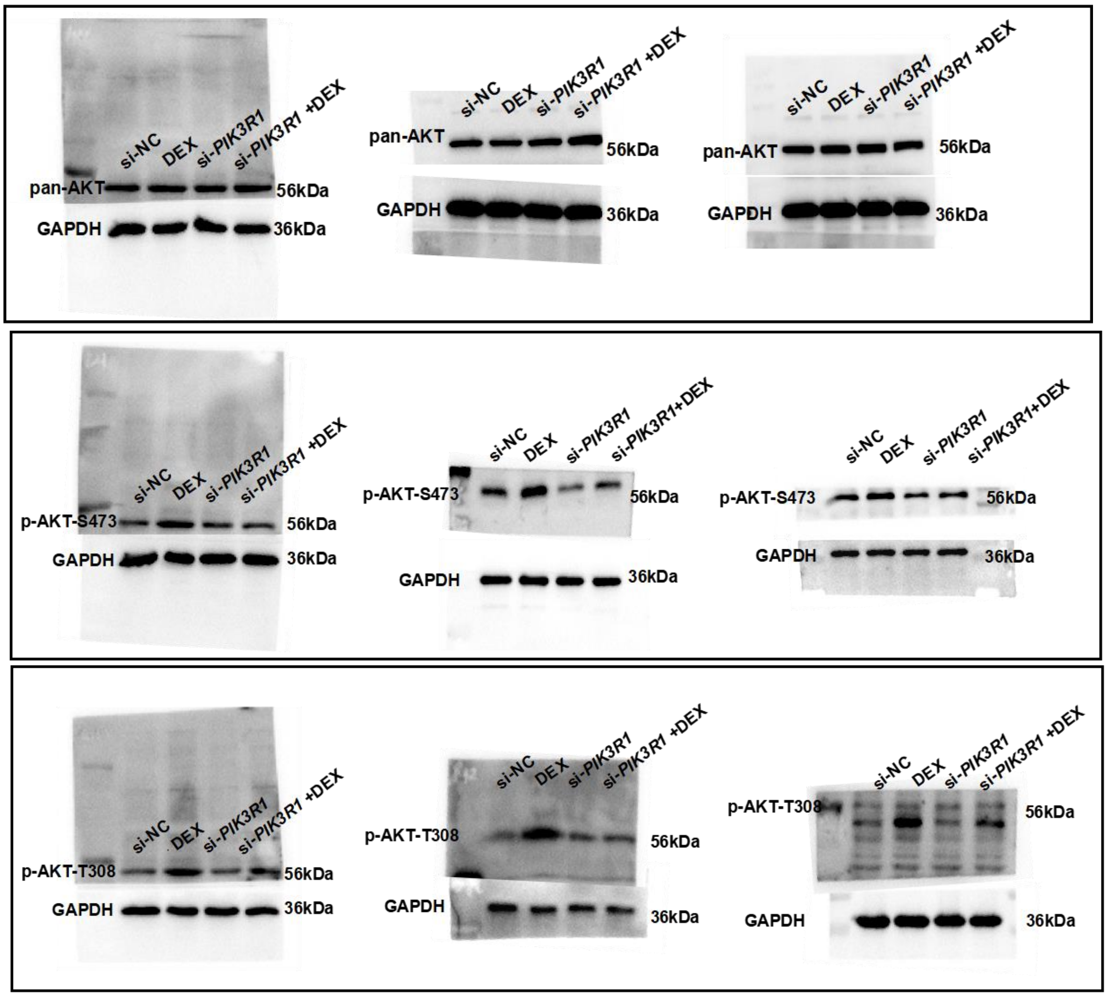

5. Figure 3A

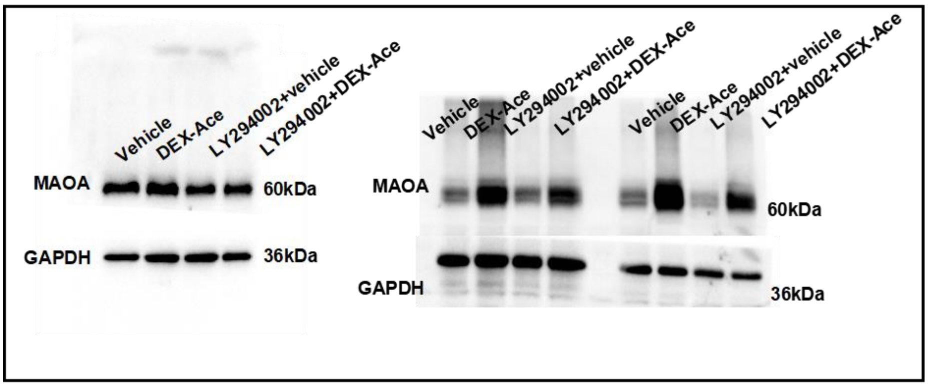

6. Figure 4A

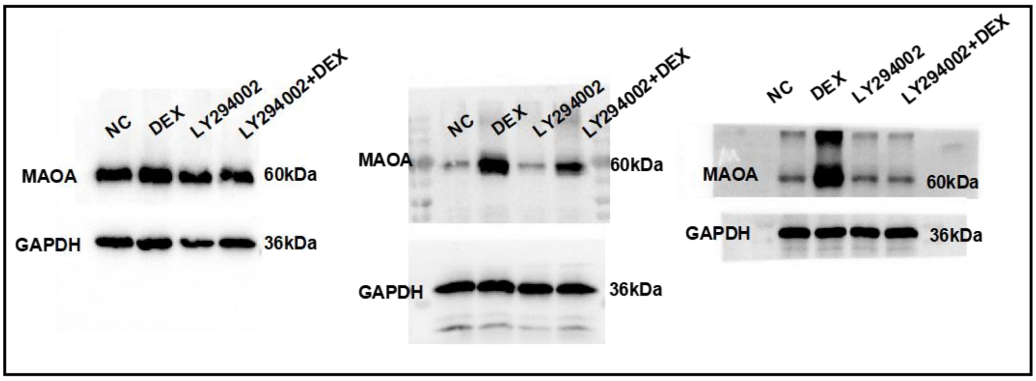

7. Figure 4B

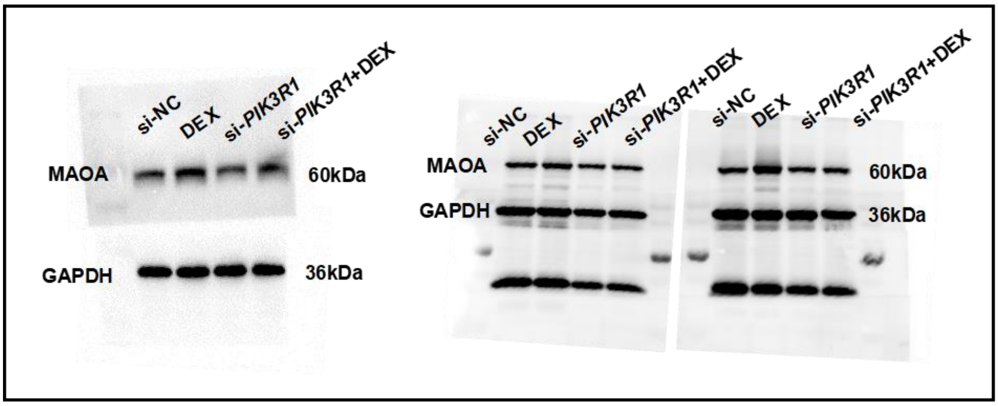

8. Figure 4C

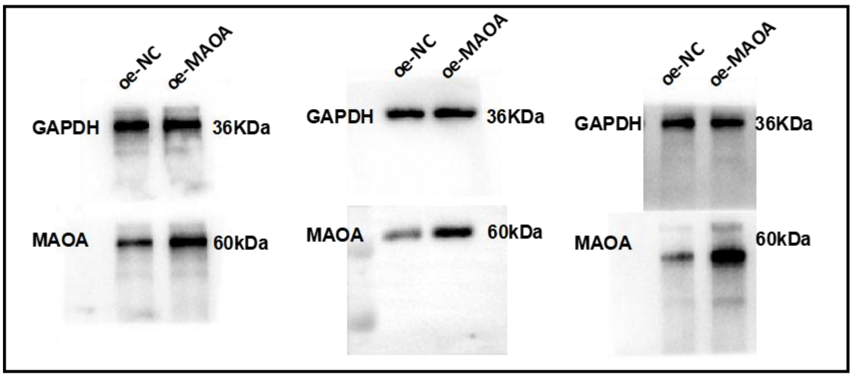

9. Figure 5C

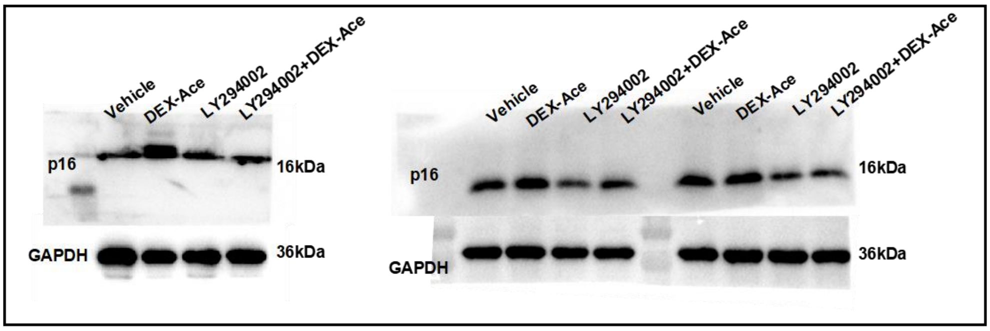

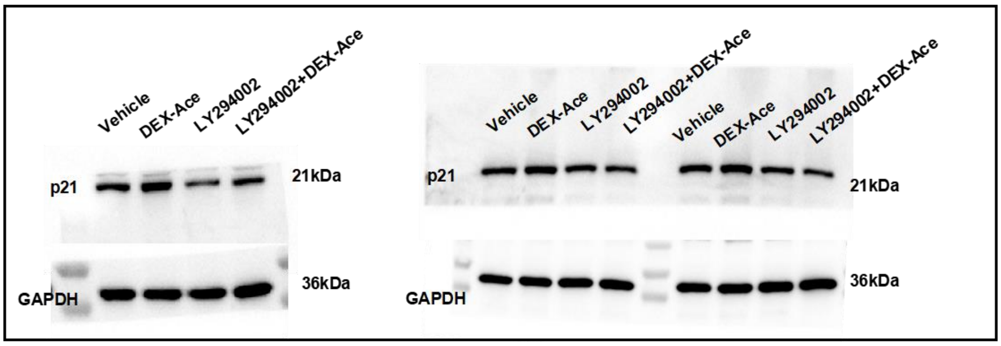

10. Figure 5E

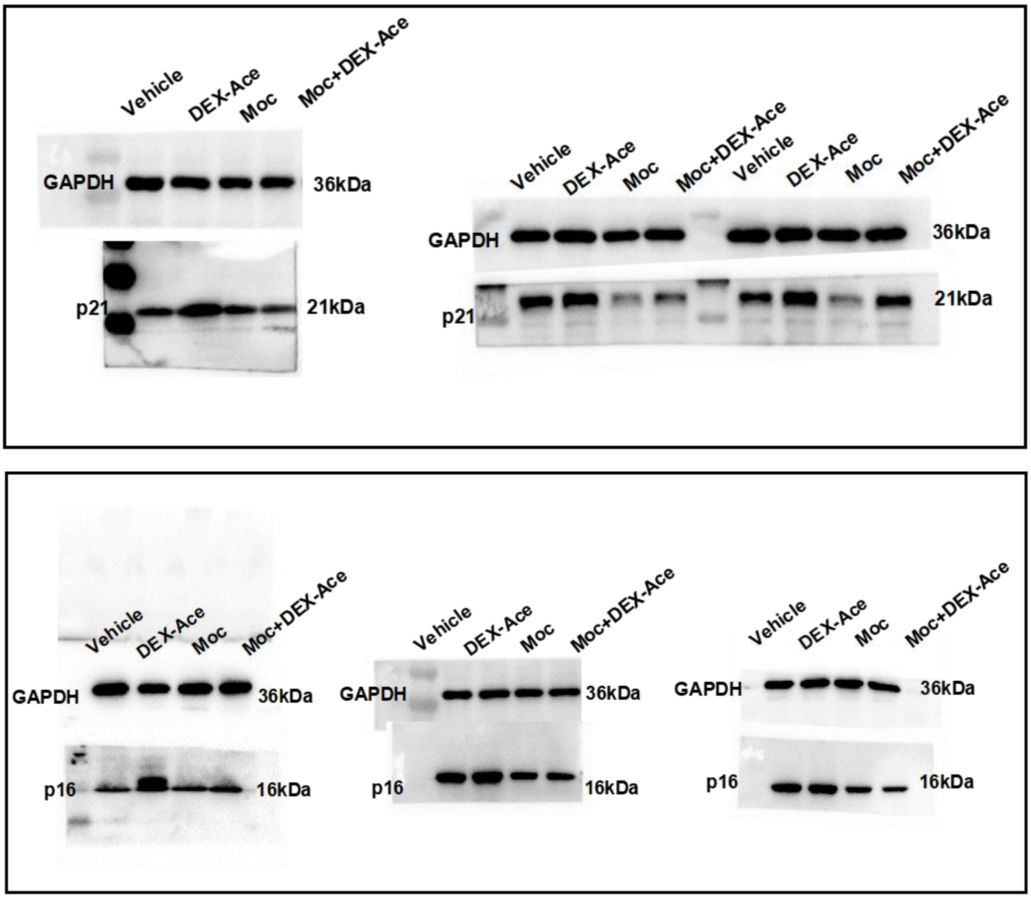

11. Figure 6C

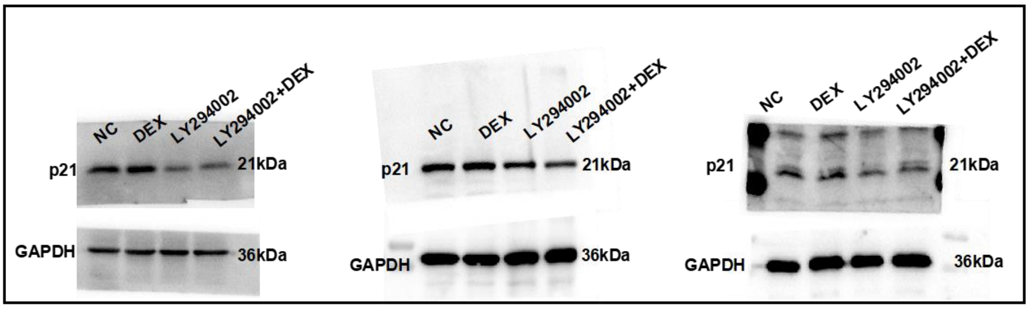

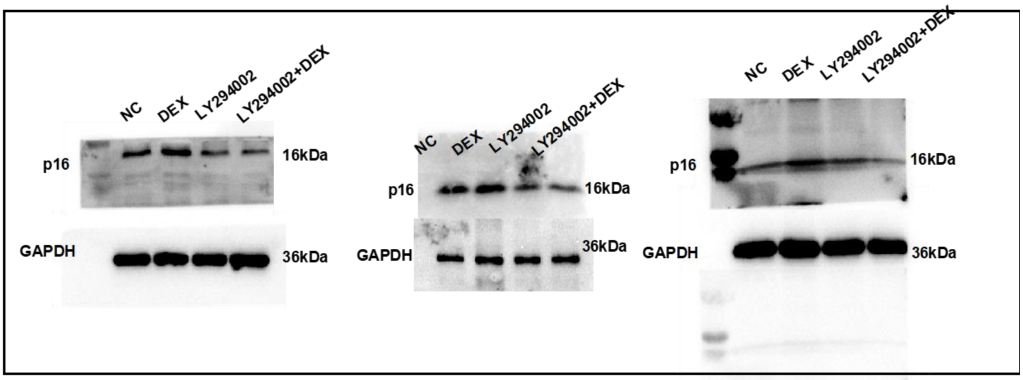

12. Figure 6F

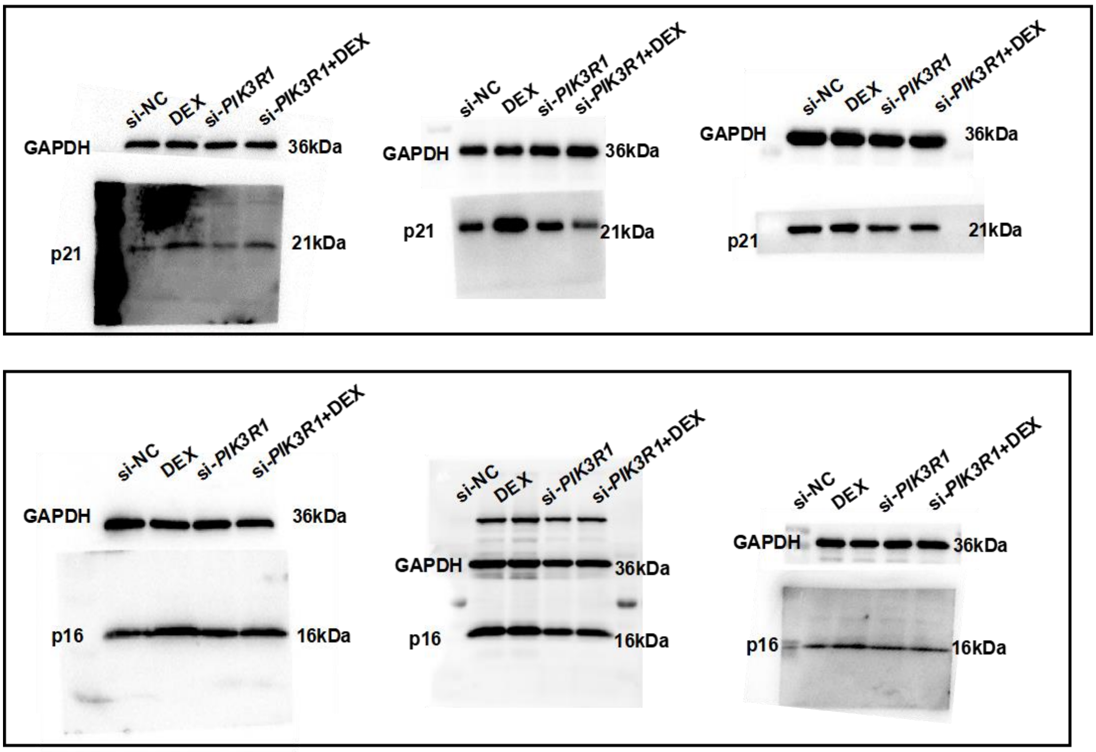

13. Figure 7A

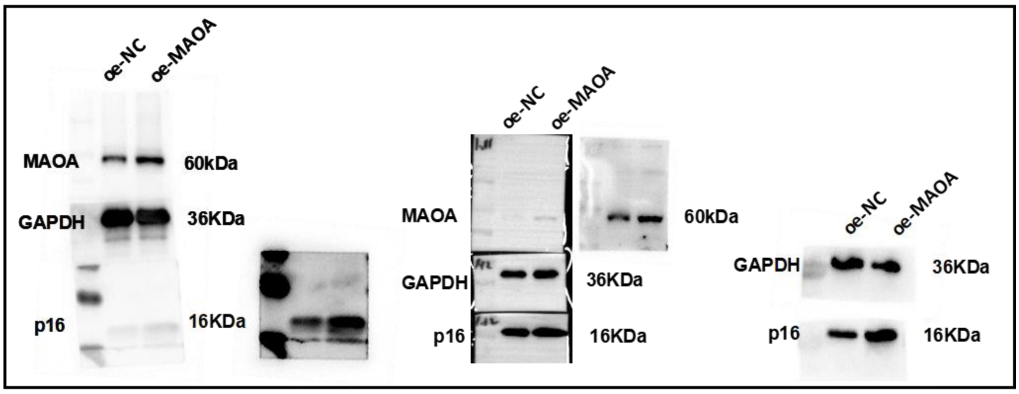

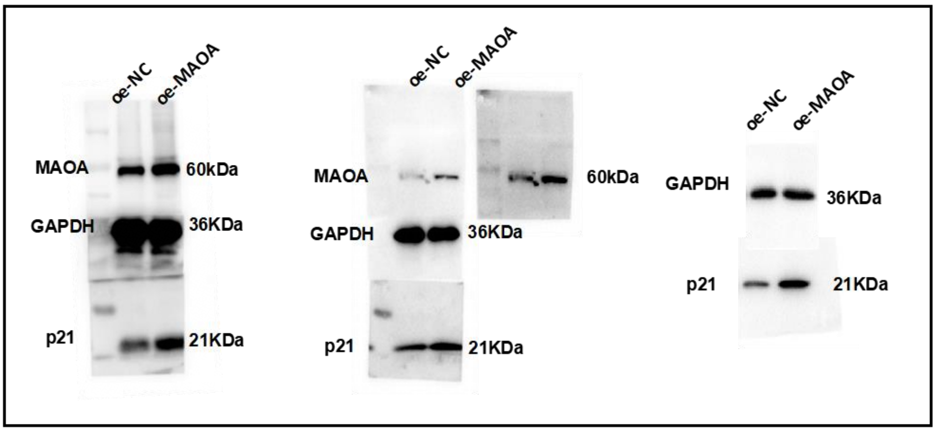

14. Figure 7E

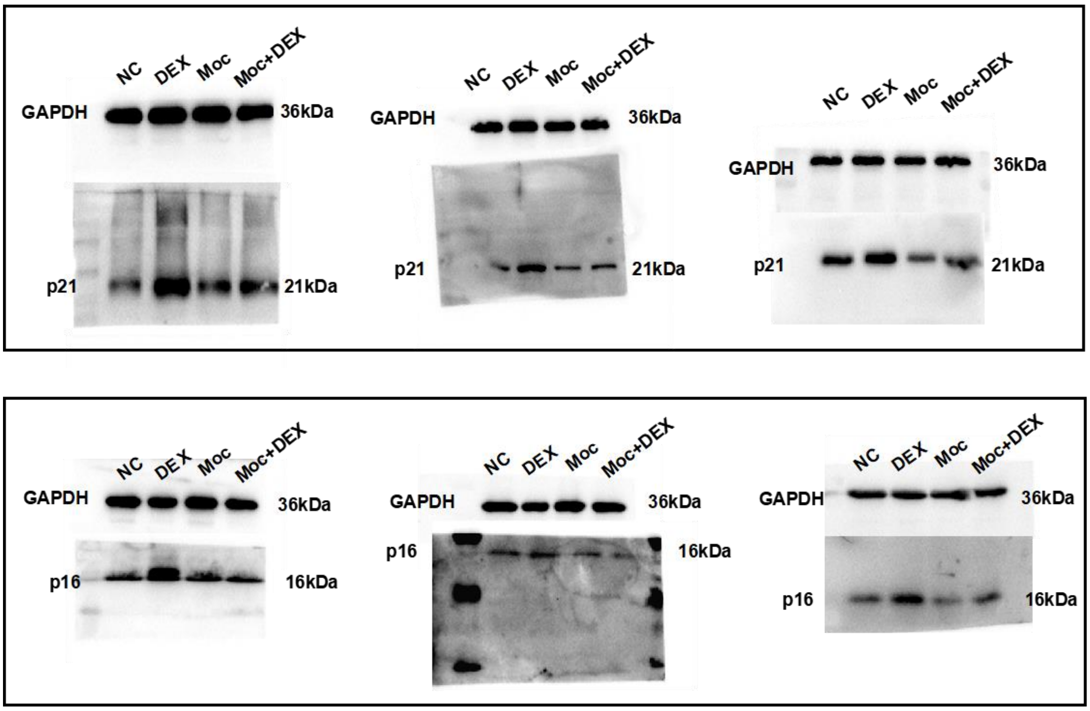

15. Supplementary Figure 1C

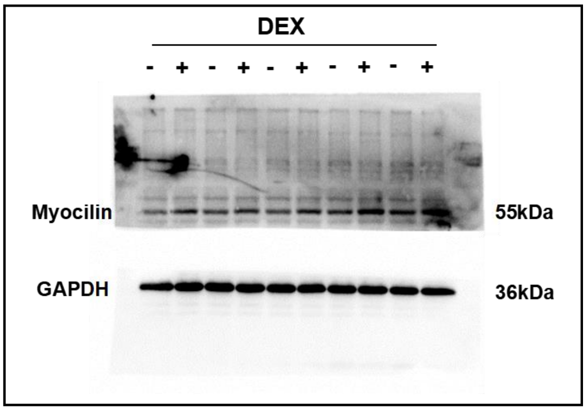

16. Supplementary Figure 2A

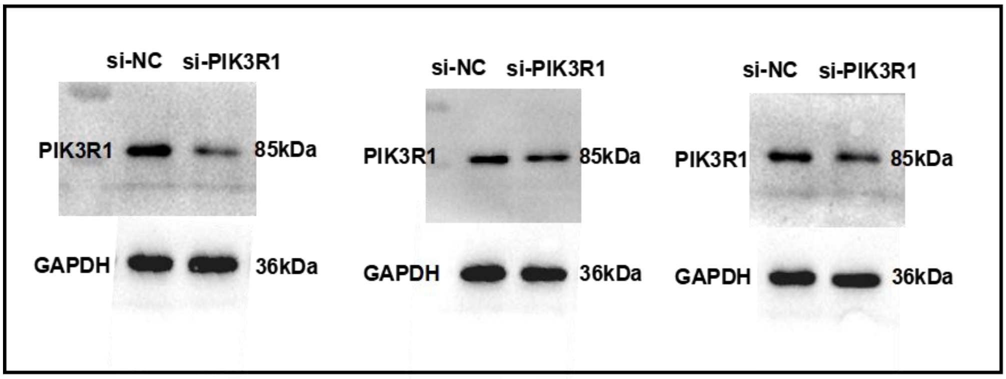

17. Supplementary Figure 3B

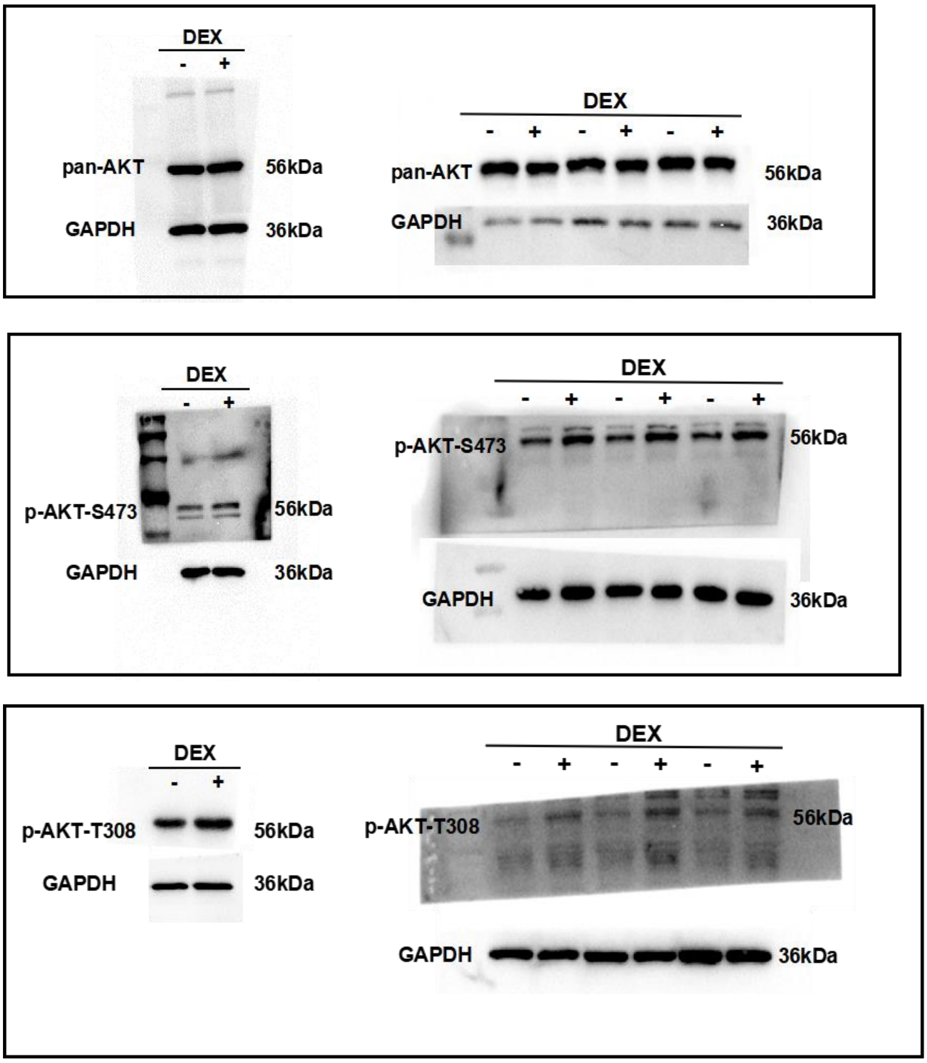

18. Supplementary Figure 3E

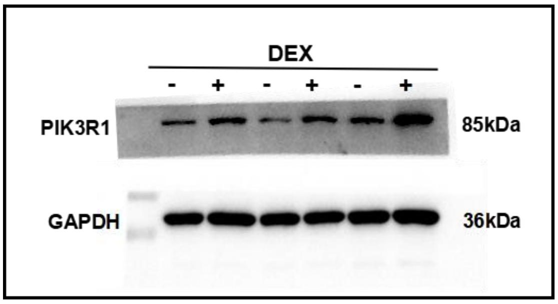

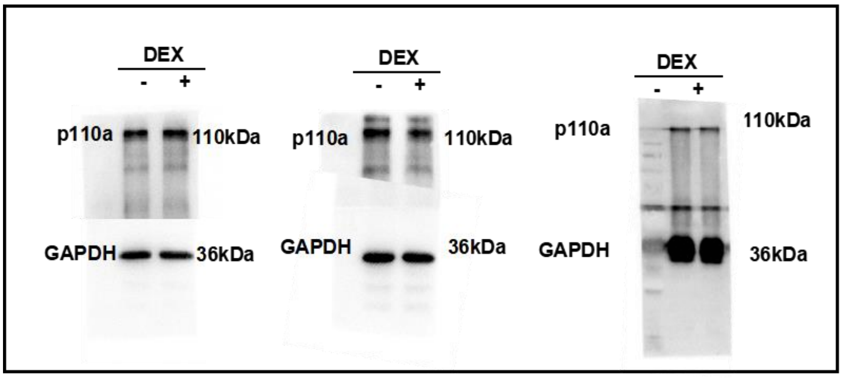

19. Supplementary Figure 6A

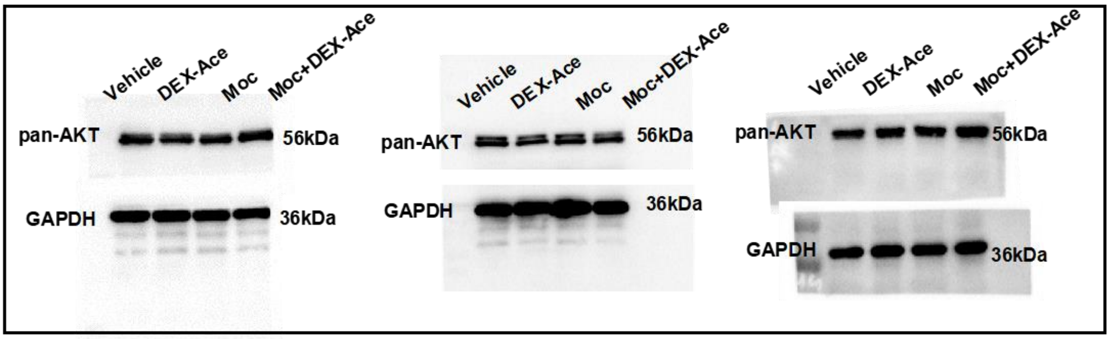

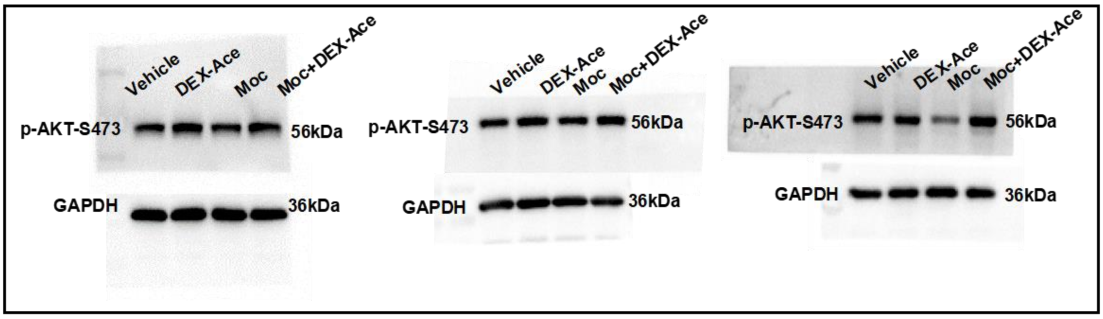

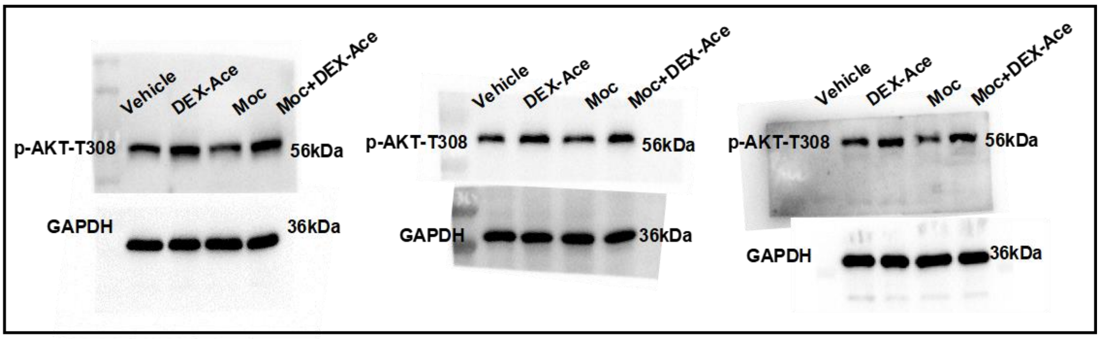

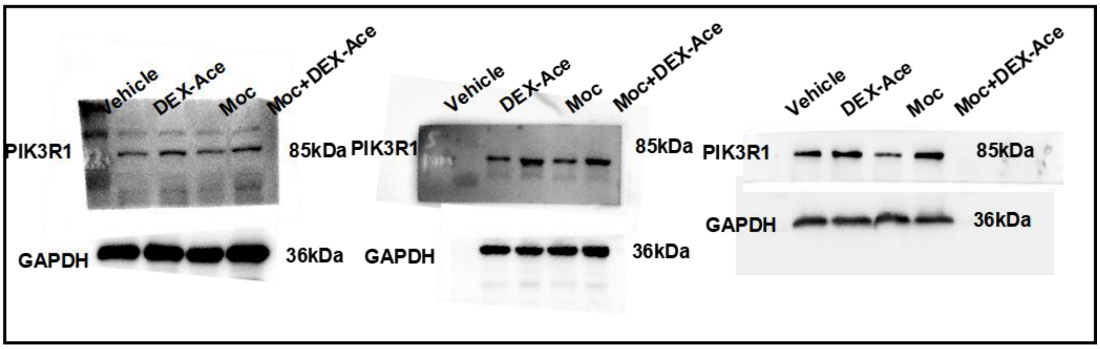

20. Supplementary Figure 6B

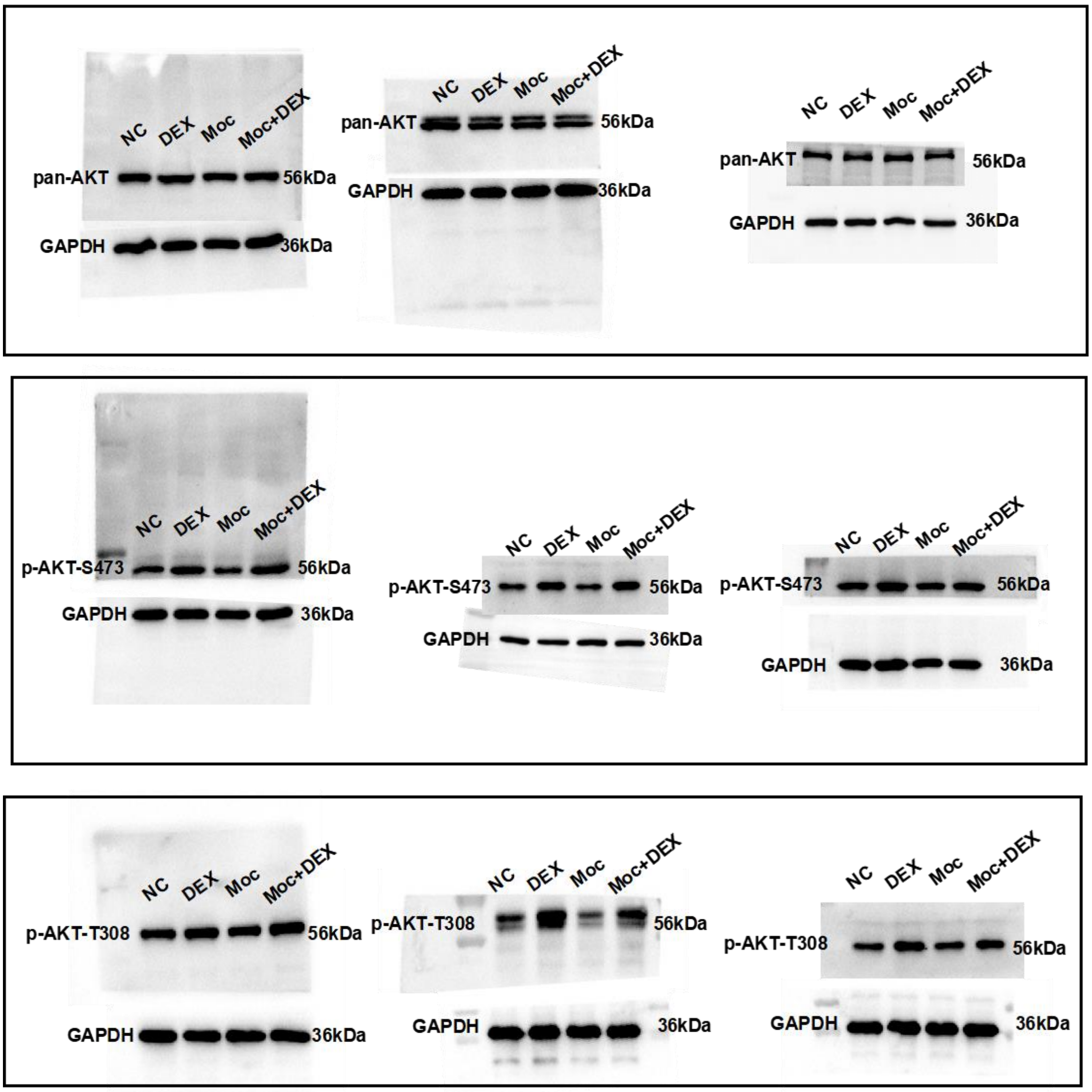

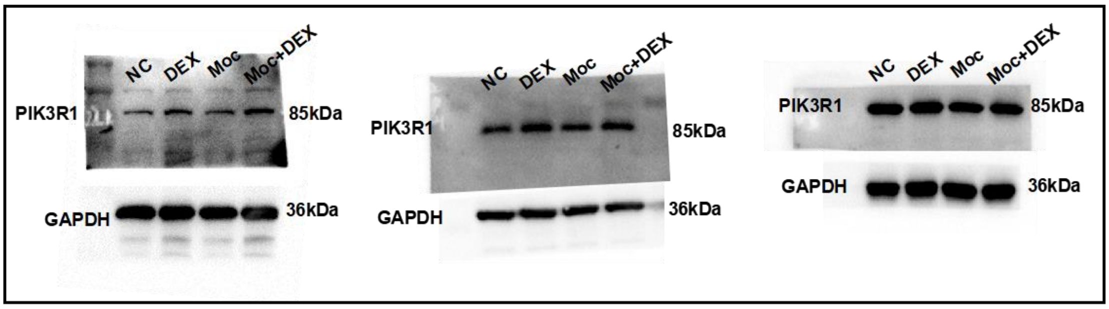

21. Supplementary Figure 7C

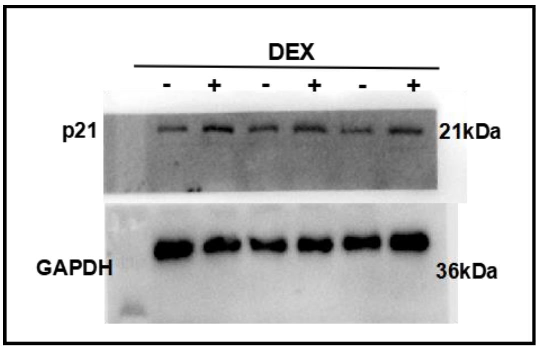

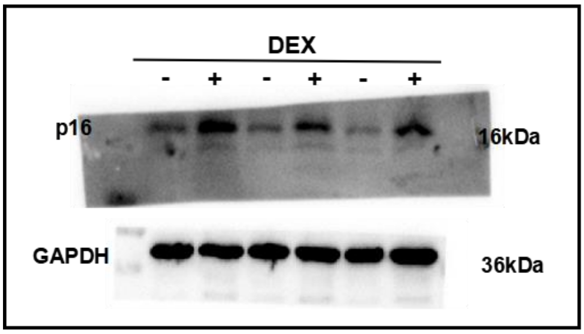

Supplement: Supplementary file 1 — Data S1: [file ACEL-24-e14452-s001.zip › revised clean copy-Supplementary materials 2-Uncropped Western Blot images.pdf]
